# TAK1 inhibition leads to RIPK1-dependent apoptosis in immune-activated cancers

**DOI:** 10.1038/s41419-024-06654-1

**Published:** 2024-04-17

**Authors:** Helene Damhofer, Tülin Tatar, Benjamin Southgate, Scott Scarneo, Karl Agger, Daria Shlyueva, Lene Uhrbom, Gillian M. Morrison, Philip F. Hughes, Timothy Haystead, Steven M. Pollard, Kristian Helin

**Affiliations:** 1 Division of Cancer Biology, The Institute of Cancer Research, London, UK; 2 Cell Biology Program, Memorial Sloan Kettering Cancer Center, New York, NY USA; 3 Biotech Research and Innovation Centre, University of Copenhagen, Copenhagen, Denmark; 4 Centre for Regenerative Medicine, University of Edinburgh, Edinburgh, UK; 5 Department of Pharmacology and Cancer Biology, Duke University School of Medicine, Durham, NC USA; 6 EydisBio Inc., Durham, NC USA; 7 Department of Immunology, Genetics and Pathology, Uppsala University, Uppsala, Sweden

**Keywords:** CNS cancer, Targeted therapies

## Abstract

Poor survival and lack of treatment response in glioblastoma (GBM) is attributed to the persistence of glioma stem cells (GSCs). To identify novel therapeutic approaches, we performed CRISPR/Cas9 knockout screens and discovered TGFβ activated kinase (TAK1) as a selective survival factor in a significant fraction of GSCs. Loss of TAK1 kinase activity results in RIPK1-dependent apoptosis via Caspase-8/FADD complex activation, dependent on autocrine TNFα ligand production and constitutive TNFR signaling. We identify a transcriptional signature associated with immune activation and the mesenchymal GBM subtype to be a characteristic of cancer cells sensitive to TAK1 perturbation and employ this signature to accurately predict sensitivity to the TAK1 kinase inhibitor HS-276. In addition, exposure to pro-inflammatory cytokines IFNγ and TNFα can sensitize resistant GSCs to TAK1 inhibition. Our findings reveal dependency on TAK1 kinase activity as a novel vulnerability in immune-activated cancers, including mesenchymal GBMs that can be exploited therapeutically.

## Introduction

Glioblastoma multiforme (GBM) is the most common and malignant form of primary adult brain tumors with a dismal prognosis [1]. Therapeutic options are limited as GBMs are highly infiltrative to the surrounding normal brain parenchyma precluding complete surgical removal. Moreover, GBMs are often detected late, display substantial inter- and intra-tumoral heterogeneity and respond poorly to most cytotoxic treatment regimens [2, 3].

Compelling evidence suggests that therapy resistance and subsequent tumor recurrence in GBM is attributed to the persistence of a population of glioblastoma stem cells (GSCs) within patient tumors. Elimination of these plastic tumor-initiating cells is considered key to achieving long-lasting therapeutic success [4]. Unfortunately, therapies targeting GSCs have been elusive, and are hampered by genetic heterogeneity. A deeper understanding of the molecular pathways maintaining GSC survival is therefore required to find novel vulnerabilities that can be exploited therapeutically either across or within the distinct tumor subtypes.

Large-scale genomics and transcriptomics analyses have led to the classification of GBM tumors and GSCs into three main molecular subtypes: classical, proneural, and mesenchymal [5, 6]. Patients with the mesenchymal subtype have the poorest survival rates, display increased immune cell infiltration, and show the strongest degree of resistance to chemo- and radiation therapy making it the subtype with the most pressing need for additional treatment options [6, 7]. However, multiregional sampling and single-cell sequencing studies have shown that individual GBM tumors contain mixtures of tumor cells from multiple transcriptional subtypes and cellular states display a high degree of plasticity with the most prominent example of a proneural-to-mesenchymal transition occurring in 59% of patients upon disease relapse following therapy [6, 8]. This phenotypic plasticity in GBM is thought to be one of the main drivers of failure to respond to current treatment modalities.

Both cell intrinsic factors such as mutations in tumor suppressors *NF1* and *PTEN* as well as external stimuli from the tumor microenvironment contribute to the acquisition of mesenchymal subtype and stem cell features in GBM [5, 6, 9]. One of the key external signals regulating this transition is tumor necrosis factor alpha (TNFα), which through binding to the TNF receptor activates NF-κB and MAPK signaling pathways and consequently transcription of inflammatory and survival genes [10]. Conversely, TNFα can also trigger apoptosis via activation of Caspase-8 and receptor-interacting serine/threonine kinase 1 (RIPK1); and TGFβ activated kinase 1 (TAK1) is an important regulator in controlling cell fate outcomes upon TNF receptor stimulation [11–13].

Here using unbiased CRISPR screening, we report on the identification of TAK1 as a novel, selective dependency in GSCs and other cancers with activated immune-signaling pathway and mesenchymal subtype features. We show that a transcriptional sensitivity signature can be employed to successfully predict sensitivity to pharmacological TAK1 inhibition, suggesting this kinase may be an important therapeutic target in around half of GBM and other cancer patients.

## Results

### MAP3K7/TAK1 is a novel selective dependency in GSCs

**Fig. 1:**
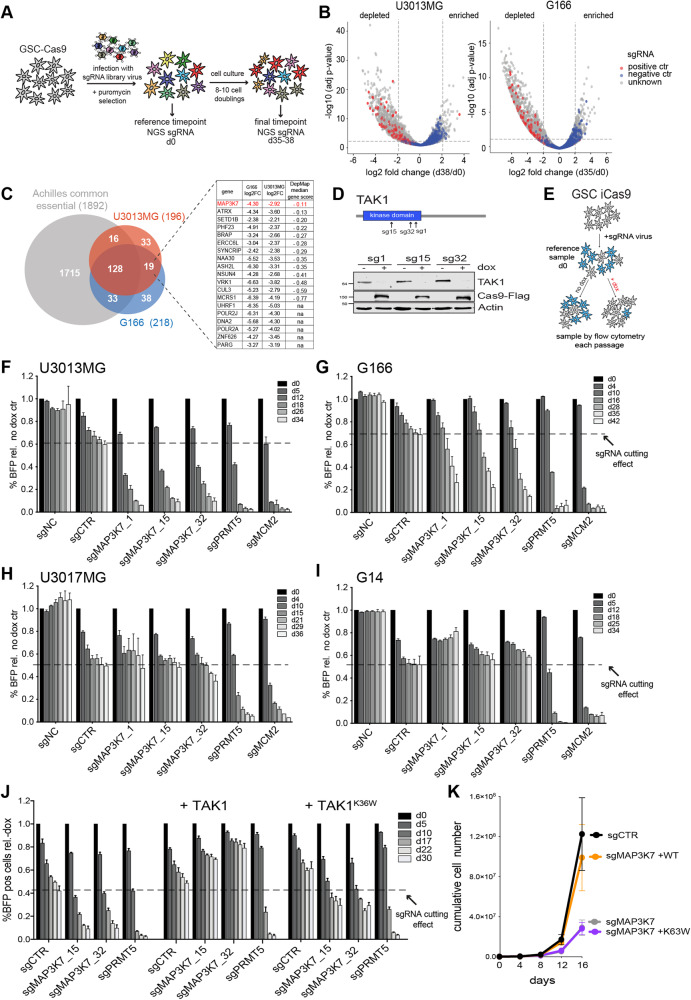
CRISPR/Cas9 knockout screens identify a MAP3K7/TAK1 dependency in a subset of GSCs. **A** Schematic of drop-out screen using a custom lentiviral sgRNA Epi-library in glioma stem cells. **B** Volcano plot representing log2 fold change and −log10 adjusted *p*-value of each sgRNA abundance comparing final (day 38 or day 35) and reference (day 0) time point in U3013MG or G166 GSC. Positive (essential genes) and negative (non-targeting) control sgRNAs are colored in red and blue, respectively. Dotted lines indicate cut-off used for hit selection. **C** Venn diagram showing overlap of hits identified in the two GSC screens and common essential genes based on DepMap data (Archilles common essential, version 22Q1). Table shows log2 fold change depletion of best sgRNA of the 19 gene hits in GSCs not essential. Ranking was performed based on the median gene dependency score of CRISPR screens from all DepMap cell lines. **D** Western blot of U3013MG iCas9 cells showing loss of TAK1 protein 72 h after doxycycline(dox)-induced expression of Cas9. **E** Cartoon depicting experimental setup of competitive growth assay in iCas9 GSCs. **F**–**I** Barplot of competitive growth assay in iCas9 GSCs. Percentage of BFP-positive cells in population was measured by flow cytometry and depicted relative to wells without Cas9 induction (- dox) at each passage. sgNC (non-targeting control sgRNA), sgCTR (targeting control sgRNA cutting outside a coding gene), sgPRMT5/sgMCM2 (essential gene positive control sgRNAs). **J** Competitive growth assay with complementation by overexpression of wild type TAK1, or catalytically inactive TAK1^K36W^ mutant. **K** Cumulative growth assay in ctr (sgCTR) and TAK1 knockout cells (sgMAP3K7) with complementation by overexpression of wild type TAK1, or catalytically inactive TAK1^K36W^ mutant.

To identify potential novel therapeutic targets in GSCs, we performed CRISPR/Cas9 drop-out screens with a focused sgRNA library targeting proteins involved in epigenetic regulation in two GSC lines, G166 and U3013MG, derived from two different GBM patients [14, 15]. A reference population of sgRNA-expressing cells was collected five days after transduction and selection (day 0) for each cell line. After continued growth for 8–10 population doublings (day 35–38), cells were harvested and the abundance of individual sgRNAs at each time point was assessed (Fig. 1A). Comparing sgRNA abundance revealed depletion of positive control genes and a slight skewing of increased abundance of negative control sgRNAs in both cell lines (Fig. 1B), which has been previously reported in several knockout screens as a feature of sgRNAs with no genomic targeting activity [16]. These data indicate good overall performance and efficiency of sgRNA depletion in the GSC screen.

To select hits with potential therapeutic impact in both GSCs we focused on genes with a minimum of two independent sgRNAs and at least 4-fold depletion compared to the day 0 reference time point. 218 genes in G166 and 196 genes in U3013MG fulfilled these criteria with 55% of genes (147) shared by both GSC lines. To exclude genes considered to be essential in most cancer cell lines, we compared the hits with a list of common essential genes identified from genome-wide CRISPR dropout screens performed in 1070 cancer cell lines (Cancer Dependency Map (DepMap), release 22Q1) and removed 177 genes considered common essential. The remaining 19 non-common essential hits identified in both GSCs were ranked based on their median DepMap gene effect score in the 1070 cell lines, with a more negative score indicating broader effects on general cellular fitness upon knockout and less selectivity (Fig. 1C). The hit with the best selectivity in GSCs was *MAP3K7*, encoding for mitogen-activated protein kinase kinase kinase 7, also known as TAK1 [17]. Most cancer cell lines (>90%) are unaffected by *MAP3K7* depletion, assessed by a DepMap gene dependency score above −0.5, in contrast to common essential gene *MCM2* (Figure S1A).

To validate *MAP3K7* as a novel GSC-selective dependency we generated GSC lines with doxycycline-inducible expression of Cas9 (iCas9) and transduced them with sgRNAs targeting the kinase domain of *MAP3K7*. Loss of TAK1 protein upon induction of Cas9 expression in GSCs was confirmed by western blot (Fig. 1D). To measure the impact of TAK1 depletion on GSC growth we performed a competitive growth assay by transducing 50% of iCas9 GSCs with different sgRNA constructs (marked by BFP expression) and measured their growth in relation to cells without sgRNA expression (Fig. 1E). The competitive growth assay confirmed depletion of cells expressing sgRNAs targeting *MAP3K7* in both U3013MG and G166 cells (Fig. 1F, G). The same *MAP3K7* targeting sgRNAs did not affect growth in immortalized human fibroblasts (BJ hTERT) or retinal pigmental epithelial cells (RPE-1 hTERT) nor in other cancer cell lines (Figure S1B).

Interestingly, when we expanded our validation experiments to test *MAP3K7* dependency in more patient-derived GSC lines, we discovered two additional GSCs, U3017MG and G14, to be unaffected by *MAP3K7* knockout (Fig. 1H, I). This was not due to ineffective protein depletion, as all four GSCs tested showed complete loss of TAK1 protein upon sgRNA expression (Figure S1C). Thus, by performing a focused CRISPR knockout screen we uncovered a novel selective dependency on *MAP3K7* gene function in a specific subset of GSCs.

### Cytoplasmatic kinase function of TAK1 is crucial for GSC survival

TAK1 is a key integrator of signaling events initiated by surface receptors that regulates different signaling pathways including NF-κB, JNK, and p38 in the cytoplasm [18]. However, TAK1 has also been reported to have potential functions in the nucleus as a component of the mammalian ADA2a-containing histone acetyltransferase (ATAC) complex [19]. We performed cellular fractionation in several GSCs and found endogenous TAK1 to be present in the cytoplasm as well as nucleoplasm and chromatin fractions (Figure S2A).

To address in which cellular compartment TAK1 exerts its pro-survival function, we ectopically expressed TAK1 fusion constructs containing an N-terminal nuclear export signal (NES::TAK1) or a triple nuclear localization signal (3xNLS::TAK1), to force localization of TAK1 to either the cytoplasm or nucleus, and confirmed their cellular localization by immunofluorescence (Figure S2B). While ectopic expression of wild-type and NES::TAK1 was fully able to rescue the growth defect caused by TAK1 knockout, nuclear localized 3xNLS::TAK1 failed to restore cell growth (Figure S2C). Although failure to rescue with the 3xNLS::TAK1 could also be due to lower protein expression compared to wild-type (Figure S2D), the rescue achieved with NES::TAK1 suggests that the cytoplasmatic function of TAK1 is the main contributor to the loss-of-function phenotype observed.

We also tested if the catalytic activity of TAK1 was required for its pro-survival function and found that a catalytically inactive mutant (TAK1^K63W^) [18] was not able to restore growth (Fig. 1J). We confirmed the dependency on catalytically active TAK1 in cumulative growth assays (Fig. 1K), and our results also showed that expression of TAK1^K63W^ had a dominant-negative effect on cell growth (Figure S2E). These results indicate that GSCs depend on the cytoplasmic, but not nuclear, kinase function of TAK1 for cell survival.

### TAK1 depletion leads to induction of RIPK1-dependent apoptosis via TNFR1

**Fig. 2:**
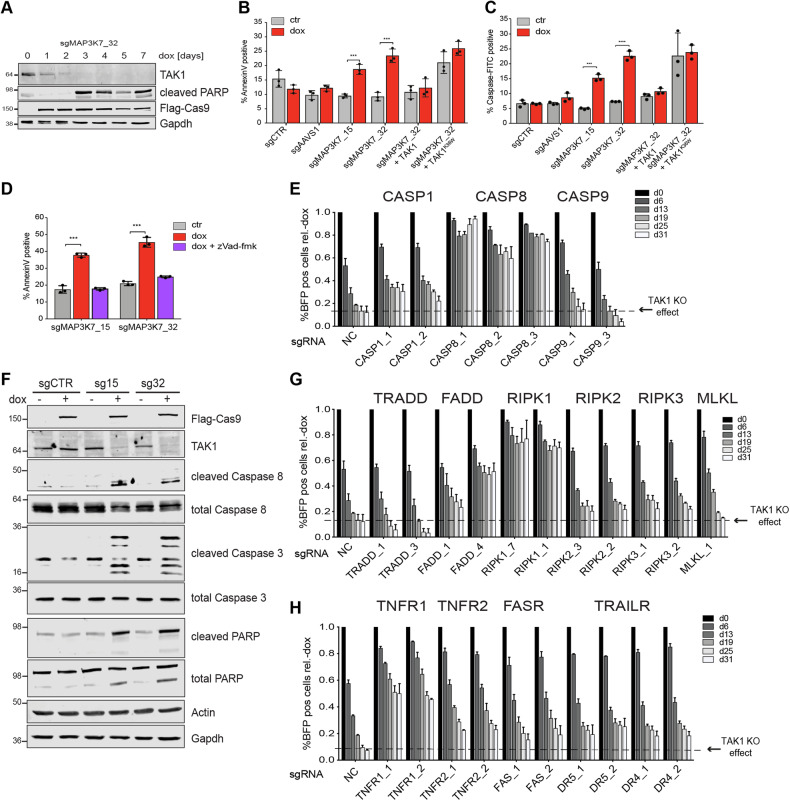
*MAP3K7* deletion leads to induction of RIPK1-dependent apoptosis via TNFR1 signaling. **A** Representative western blots for the indicated proteins of time course experiment of sgMAP3K7_32 expressing U3013MG iCas9 cells upon induction of Cas9 by dox treatment for up to 7 days. **B**–**D** Barplot of % Annexin V positive cells (**B** and **D**) or Caspase-FITC cells (**C**) quantified by flow cytometry 4 days after induction of TAK1 knockout (dox). **E** Competitive growth assays showing %TAK1 knockout cells over time in the population (measure by BFP abundance) in the presence of a second sgRNA targeting Caspases-1, -8, and -9. sgRNA including gene name is shown on the x-axis. Percentage of BFP-positive cells in population was measured by flow cytometry and depicted relative to wells without Cas9 induction (- dox) at each passage. Dotted line indicates the effect of TAK1 depletion on the population in the presence of a second non-targeting sgRNA (NC). Error bar indicating mean + SD for 3 biological replicates at each time point. **F** Western blots of different apoptosis markers 4 days after induction of Cas9 expression with doxycycline (dox) in sgCTR, sgMAP3K7_15 and sgMAP3K7_32 expressing cells. **G**, **H** Competitive growth assay as (**E**) with second sgRNAs targeting different apoptosis complex members (**G**) or death receptor genes (**H**).

To investigate the phenotypic responses following ablation of *MAP3K7*, we harvested sgMAP3K7 expressing cells at different times after knockout induction. TAK1 protein levels were depleted 3 days after induction of Cas9 expression, which coincided with PARP cleavage (Figs. 2A and S3A), increased Annexin V positivity (Fig. 2B), Caspase activation (Fig. 2C), and an increased Sub-G1 cell cycle population (Figure S3B), all known markers of apoptosis. Annexin V induction and Caspase activation were completely reversed by expression of wild type but not catalytic inactive TAK1^K63W^. In addition, TAK1^K63W^ expressing cells displayed increased apoptosis even before induction of TAK1 knockout (Fig. 2B, C), suggesting a dominant-negative effect of catalytic inactive TAK1. Induction of apoptosis could be completely reversed by treatment with pan-Caspase inhibitor zVad-fmk (Fig. 2D). These data indicate that TAK1 kinase activity protects U3013MG cells from a caspase-dependent cell death.

To determine which Caspase-dependent death pathway is triggered by TAK1 depletion, we expressed a second sgRNA targeting initiator Caspases-1, -8, and -9, key mediators of either the inflammatory, extrinsic, and intrinsic apoptosis pathway, respectively [20] (Figure S3C). Whereas TAK1 knockout led to depletion of cells in the presence of sgRNAs targeting Caspase-1 and Caspase-9, the TAK1 phenotype was completely reverted by simultaneously removing Caspase-8 (Fig. 2E). Western blotting confirmed the activation of the extrinsic apoptotic signaling cascade measured by cleavage of initiator Caspase-8 and effector Caspase-3 as well as PARP upon TAK1 knockout in U3013MG and G166 cells (Figs. 2F and S3D).

Caspase-8 has been shown to form complexes with different adaptor proteins such as receptor-interacting serine/threonine kinases (RIPK1-3), FAS associated via death domain (FADD), and TNF receptor type 1-associated DEATH domain (TRADD) to convert signals received from different death receptors to activate downstream executioner Caspases-3/7 and ultimately lead to induction of cell death [21]. We designed sgRNAs targeting each of these mediators and found both knockout of RIPK1 and FADD as well as TNF receptor 1 could protect GSCs from growth defects caused by loss of TAK1 function (Figs. 2G, H and S3C). Based on these results, we conclude that in sensitive GSCs loss of TAK1 triggers TNFR1-mediated Caspase-8/RIPK1/FADD complex activation thus resulting in apoptotic cell death.

### TAK1 depletion leads to apoptosis in GSCs with constitutive TNFR signaling

**Fig. 3:**
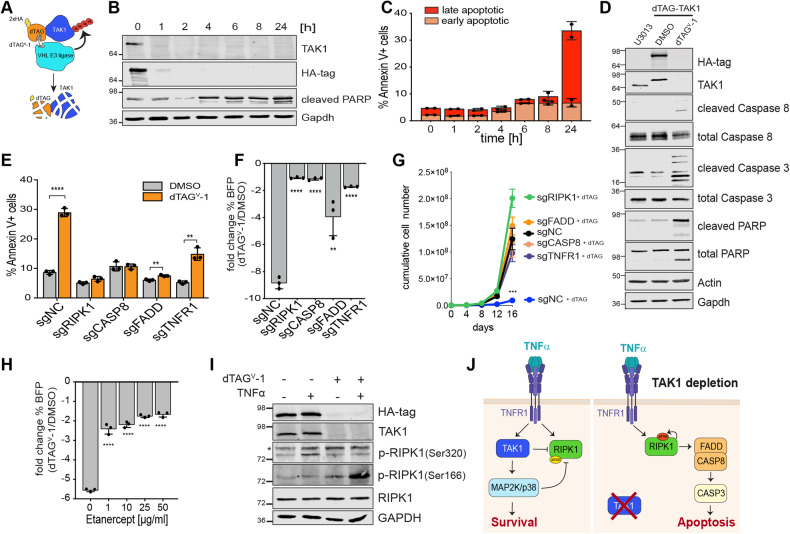
TAK1-degradation leads to RIPK1-dependent apoptosis. **A** Schematic cartoon of TAK1 depletion using a dTAG-TAK1 degradation system. **B** Western blots of time course experiment treating dTAG-TAK1 GSCs with 100 nM dTAG^V^-1 ligand for indicated amount of time. **C** Barplot of total % Annexin V positive cells quantified by flow cytometry after treatment with 100 nM dTAG^V^-1 ligand. 2 biological replicates at each time point are shown. Early apoptotic cells are defined as Annexin V + /DAPI- and late apoptotic cells as Annexin V + /DAPI+. **D** Western blot of apoptosis markers 24 h after treatment with 100 nM dTAG^V^-1 ligand. **E** Barplot of total % Annexin V positive cells quantified by flow cytometry after treatment with 100 nM dTAG^V^-1 ligand for 4 days in dTAG-TAK1 degron cells after knockout of indicated gene. **F** Barplot of competitive growth assay of dTAG-TAK1 cells expressing BFP and parental GSCs. Fold change of %BFP-positive cells in population after treatment with dTAG^V^-1 ligand for 7 days is shown relative to DMSO-treated control. **G** Cumulative growth assay in dTAG- TAK1 degron cells upon knockout of the second indicated gene by CRISPR and treatment with dTAG^V^-1 ligand. **H** Barplot depicting fold change of %BFP-positive dTAG-TAK1 cells in population after treatment with dTAG^V^-1 ligand for 7 days relative to DMSO-treated control and treatment with increasing concentrations of TNF ligand blocking antibody Etanercept. **I** Western blot of RIPK1 phosphorylation events after treatment with TNFα with or without TAK1 protein depletion. *denotes unspecific band. **J** Cartoon of molecular response to TAK1 inhibition in TAK1-dependent GSCs.

To explore the mechanisms of TAK1 sensitivity further we generated a chemical-inducible TAK1 protein degradation system [22] by ectopic expression of a dTAG-TAK1 fusion construct in cells in which we subsequently knocked out endogenous *MAP3K7*. This approach enables the acute depletion of TAK1 protein upon treatment with the heterobifunctional ligand dTAG^V^-1 [23] (Fig. 3A).

Assessment of the degradation kinetics in the dTAG-TAK1 cells confirmed near complete loss of dTAG-TAK1 one hour after treatment with dTAG^V^-1, followed by robust induction of PARP cleavage at four hours (Fig. 3B), and a gradual increase of first early and then late apoptotic cells (Fig. 3C). As described for TAK1 knockout, TAK1 degradation led to activation of the extrinsic apoptotic cascade measured by cleavage of Caspase-8 and Caspase-3 (Fig. 3D). Moreover, the induction of apoptosis and reduced cell growth was dependent on activation of the Caspase-8/RIPK1/FADD complex via TNFR1 (Fig. 3E–G).

We hypothesized that TNFR1 was constitutively activated in GSCs by the presence of TNFR1 ligands, such as TNFα or Lymphotoxin A. To test this, we repeated the competitive growth assay in the presence of increasing concentrations of Etanercept, a chimeric decoy receptor binding to and inhibiting soluble TNFα [24]. and found that blocking TNFα strongly mitigated the effect caused by TAK1 depletion (Fig. 3H).

The pro-apoptotic function of RIPK1 is tightly controlled by several kinases, including TAK1 and its substrates IKKα/β, TBK1 and p38/MK2 which phosphorylate and inhibit RIPK1 activity in response to TNFα [25–28]. The resulting S320 phosphorylation of RIPK1 has been reported to prevent its association with FADD/Caspase-8, thereby inhibiting the induction of RIPK1-dependent apoptosis in response to TNFR pathway activation [25, 27]. As expected, treatment of GSCs with TNFα led to a TAK1-dependent inhibitory S320 phosphorylation of RIPK1 and full activation of RIPK1 (measured by autophosphorylation on S166) only occurred in the absence of TAK1 (Fig. 3I). Interestingly, neither pharmacological inhibition of p38α/β nor knockout of TBK1, p38α, MK2, or NEMO (to inactivate the IKK complex) was able to phenocopy the effect observed with TAK1 depletion (Figure S3E, F) suggesting only removing the upstream master regulatory kinase is sufficient to shift the balance from survival towards apoptosis. Our results support a model in which TNFα secreted from GSCs leads to constitutive stimulation of TNFR1. In the presence of TAK1, the activation of TNFR1 does not induce apoptosis, because the pro-apoptosis mediator RIPK1 is kept inactive by TAK1 kinase activity via RIPK1 S320 phosphorylation [25, 29]. However, when TAK1 is inactivated in this context, RIPK1 becomes active and induces apoptosis by associating with the Caspase-8/FADD complex (Fig. 3J).

### Pharmacological inhibition of TAK1 induces apoptosis in genetically sensitive GSCs

**Fig. 4:**
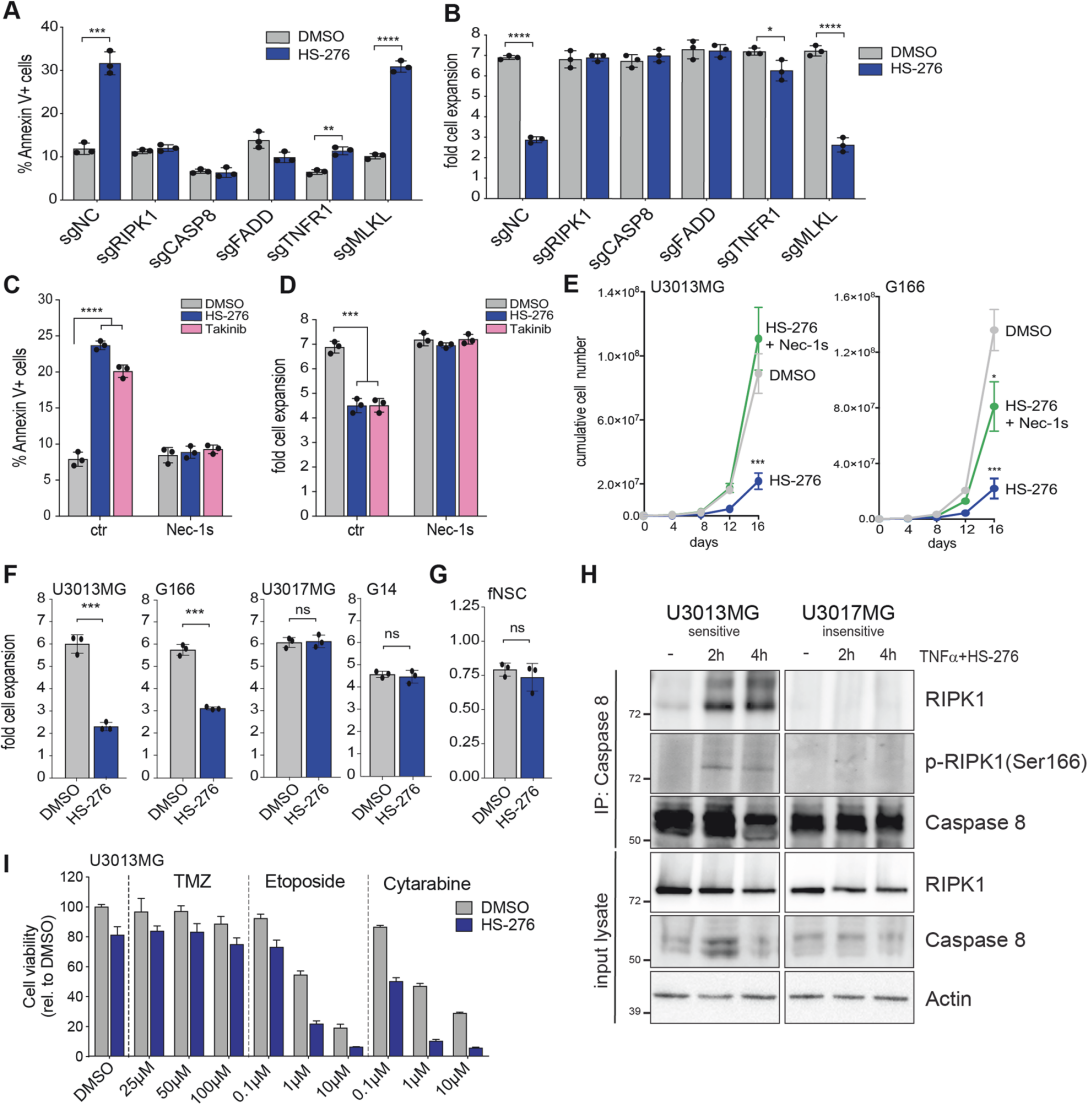
Pharmacological inhibition with novel selective TAK1 inhibitor HS-276 induces apoptosis in GSCs. **A**, **B** Barplot of %Annexin V positive cells (**A**) and fold cell expansion (**B**) of U3013MG cells with knockout of TNFR pathway members upon 4 days treatment with DMSO or 3 µM HS-276. **C**, **D** Barplot of %Annexin V positive cells (**C**) and fold cell expansion (**D**) of U3013MG cells treated for 4 days with HS-276 or Takinib in combination with RIPK1 inhibitor Necrostatin-1s (Nec-1s). **E** Cumulative growth assay of U3013MG or G166 cells treated with DMSO, HS-276, or a combination of HS-276 and Nec-1s. **F** Barplot of fold cell expansion within 4 days of treatment with DMSO or 3 µM HS-276 in 4 different glioma stem cell lines (U3013MG, G166, U3017MG, and G14). **G** Barplot of fold cell expansion within 4 days of treatment with DMSO or 3 µM HS-276 in fetal neural stem cells (fNSC, U5). **H** Western blot of death complex IIb formation in GSCs upon treatment with TNFα and HS-276 for 2 or 4 h. **I** Barplot of cell viability relative to DMSO in U3013MG cells treated with indicated chemotherapeutic drugs in increasing concentrations alone or in combination with HS-276 for 4 days.

TAK1 is known to play a critical role in mediating TNF signal transduction and downstream NF-κB activation, helping to sustain pro-inflammatory signaling in diseases including rheumatoid arthritis, irritable bowel disease and lupus [30]. Pharmacological inhibition of TAK1 is considered an attractive strategy to treat inflammatory diseases. Recently a novel ATP competitive TAK1 inhibitor termed HS-276 was developed. HS-276 is a highly selective potent inhibitor of TAK1 kinase activity with improved bioavailability as compared to the parental compound Takinib [31–33]. Titration of HS-276 on TAK1 knockout sensitive and insensitive GSCs revealed doses of up to 3 µM affecting cell viability only in sensitive GSCs (Figure S4A). We treated U3013MG GSCs with increasing concentrations of HS-276 and found robust induction of apoptosis at drug concentrations of 1 and 3 µM (Figure S4B), which was accompanied by reduction in cell numbers (Figure S4C). 1–3 µM is consistent with concentrations required to achieve ATP competitive inhibition of TAK1 in cells [32]. HS-276 effectively inhibited phosphorylation of known downstream kinase targets of TAK1 such as p38, p65, and JNK (Figure S4D). By performing cumulative growth assays, we found HS-276 led to a strong reduction in cell numbers, with effects similar to TAK1 depletion (Figure S4E). Knockout of RIPK1, Caspase-8 and FADD resulted in a complete, or in the case of TNFR1 near complete rescue of the HS-276 induced phenotype in U3013MG (Fig. 4A, B) and G166 cells (Figure S4F). These results precisely recapitulate the genetic dependencies found in the TAK1 knockout and dTAG degradation models and confirm on-target inhibitory activity of HS-276 on TAK1 function in GSCs.

To further assess the dependency on RIPK1 function for apoptosis induced by TAK1 inhibition, we combined HS-276 or Takinib treatment with the RIPK1 kinase inhibitor Necrostatin-1s (Nec-1s). Nec-1s reversed both induction of apoptosis as well as reduction in cell numbers (Fig. 4C, D), confirming activation of RIPK1 kinase activity as a key event to cause apoptosis in the absence of TAK1. Co-administration of Nec-1s with HS-276 completely reversed the effects on long-term cell growth caused by TAK1 kinase inhibition in three GSC lines (Figs. 4E and S4G). Importantly, when testing HS-276 on the four GSC cell lines we previously established to be either sensitive (G166, U3013MG) or insensitive (U3017MG, G14) to knockout of TAK1 (Fig. 1F–I) we found HS-276 treatment affected only sensitive but not insensitive GSCs (Fig. 4F). Moreover, three different fetal neural stem cell lines (fNSCs), the closest healthy untransformed equivalent to GSCs, were unaffected by exposure to HS-276 (Figs. 4G and S4H). Treatment of TAK1-sensitive U3013MG cells with HS-276 in combination with TNFα resulted in robust activation (S166 phosphorylation) and recruitment of RIPK1 to death complex IIb as evidenced by co-precipitation with Caspase 8 (Fig. 4H). Since RIPK1 was not activated nor recruited to death complex IIb in insensitive U3017MG cells, this agrees with our model that TAK1 inhibition only triggers RIPK1-dependent apoptosis in sensitive GSCs (Fig. 3J).

Interestingly, co-treatment with HS-276 substantially enhanced the cytotoxic effect of chemotherapeutic drugs Etoposide and Cytarabine in sensitive but not insensitive cells, whereases the main therapeutic drug used in GBM patients, temozolomide (TMZ), displayed no cooperativity (Figs. 4I and S4I). The latter result was expected because many GSCs, including U3013MG, are inherently resistant to TMZ treatment due to high expression of *MGMT* (Figure S4J) [34]. Altogether, these results provide a pre-clinical proof of concept for the use of HS-276, potentially in combination with other cytotoxic drugs, for the selective targeting of TAK1-sensitive GSCs.

### TAK1-dependent GSCs express a distinct immune activation signature

**Fig. 5:**
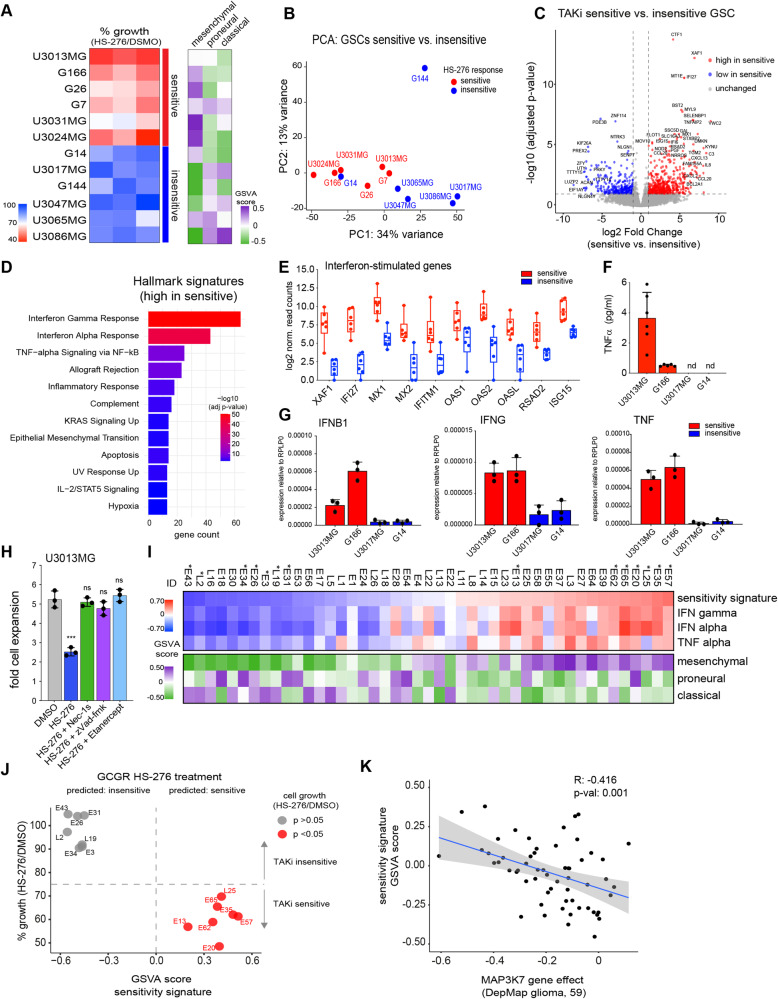
TAK1 inhibitor sensitive GSCs are characterized by high cytokine/interferon signaling gene expression signatures. **A** Heatmap showing % growth inhibition expressed as the relative reduction in cell numbers after 4 days of treatment with HS-276 relative to mean of DMSO-treated controls in 12 GSC lines. GSCs are classified as sensitive (red) or insensitive (blue) based on a significant difference between cell numbers in HS-276 and DMSO treatment conditions. Shown are representative results of 3 biological replicates. Heatmap to the right shows GSVA score of gene expression signature from cell line for mesenchymal, proneural, or classical GBM subtype. **B** Principal component analysis plot (PCA) of RNAseq data from GSC lines. HS-276 sensitive lines are shown in red, insensitive ones in blue. **C** Volcano plot of differentially expressed genes between sensitive and insensitive GSC lines (*n* = 6 in each group). Significantly higher expressed genes in sensitive GSCs are colored in red, lower expressed genes in blue, and unchanged in gray. Dotted lines indicate cut-off value used to determine deregulated genes (absolute log2 Fold Change of >1 and adjusted *p*-value of <0.1). **D** Barplot of the 12 most significantly enriched Hallmark gene signatures in GSCs sensitive to HS-276 treatment (gene set high, *n* = 513). **E** Box and wiskers plot of log2 normalized read counts of baseline expression of selected interferon-stimulated genes (ISGs) in sensitive (*n* = 6) and insensitive GSCs (*n* = 6) measured by RNAseq. Whiskers show minimum and maximum values within group. Boxes indicate median, upper, and lower quartiles. **F** ELISA of TNFα concentration in 7 days conditions GSCs supernatant (6 biological replicates). nd = not detected. **G** Barplot of *IFNB1, IFNG*, and *TNF* gene expression in GSCs measured by qPCR and normalized to *RPLP0*. **H** Barplot of fold cell expansion of U3013MG treated for 4 days with indicated drugs. **I** Heatmap of GSVA scores in GCGR-GSC lines. Samples were ranked based on sensitivity signature GSVA score. ID, GCGR patient ID. * indicates GSC lines selected for testing of responsiveness to HS-276 in vitro. **J** Scatter plot of % growth in 14 GCGR GSCs after 4 days of treatment with HS-276 relative to DMSO against the sensitivity signature GSVA score. Shown is the relative mean of 3 biological replicates (HS-276/DMSO treated). GSCs with significant reduction in cell numbers upon HS-276 treatment are indicated in red. Dotted line indicates separation based on GSVA score into predicted sensitive (positive score) and predicted insensitive (negative score) GSCs and 25% in growth reduction for sensitivity to TAK inhibition by HS-276 treatment. **K** Scatter plot of *MAP3K7* gene knockout effect against sensitivity signature GSVA score from 59 DepMap glioma cell lines.

To further elucidate the differential responses to TAK1 inhibition (TAKi), we expanded our cell line panel with eight additional patient-derived GSC lines and determined their response to HS-276. Half of all the GSC lines tested responded to TAKi by displaying a significant decrease in cell number. Although there were no clear genetic features associated with response to HS-276 (Figure S5A), we observed TAKi sensitive GSCs displayed a mesenchymal GBM subtype transcriptional signature. By contrast, proneural or classical signatures were the predominant feature in insensitive cell lines (Fig. 5A). Comparison of the principal components of the baseline gene-expression profile of TAKi sensitive and insensitive GSCs revealed separation based on their dependency on TAK1 (Fig. 5B).

To identify specific gene expression signatures characteristic to TAKi sensitive cells, we performed differential gene expression analysis comparing HS-276 sensitive and insensitive cell lines and found 804 genes with an at least 2-fold significant difference between the groups (Figs. 5C and S5B). 291 of these genes were lowly expressed in the sensitive GSCs with no notable pathway enrichments. We next examined the list of 513 genes highly expressed in TAKi sensitive lines and found a striking overlap with MSigDB Hallmark signatures characteristic of immune activation, including Interferon Gamma and Alpha response as well as TNFα signaling via NF-κB (Fig. 5D). Similar results were obtained by performing Gene set enrichment analysis (Figure S5C), which revealed high activation of immune signaling pathways specifically in GSCs sensitive to TAKi treatment.

Interferons (IFNs) constitute a family of cytokines that are fundamental modulators of both innate and adaptive immune responses and have important roles in immunosurveillance for malignant cells. The two main classes are type I IFNs, with many ligand members including IFNα and IFNβ, and type II IFNs, with IFNγ being the only known ligand [35]. Both type I and II IFNs lead to activation of the JAK/STAT signaling pathway and transcriptional upregulation of a specific set of interferon-stimulated genes (ISGs) such as *MX1*, *IFI27*, or *OASL* [36]. We examined several known ISGs in the baseline RNAseq gene expression data and observed a much higher expression of ISGs in TAKi sensitive compared to insensitive cell lines (Fig. 5E). qPCR confirmed these observations from the RNAseq data, with *IFI27* displaying 1000-fold higher expression in the TAKi sensitive compared to insensitive cell lines (Figure S5D). To address the possibility of constitutive activation of these inflammatory pathways via autocrine ligand production and subsequent receptor stimulation we measured ligand expression and found both type I and II IFN ligands *IFNB1* and *IFNG* as well as TNFR ligand *TNF* expressed in sensitive with nearly undetectable levels in insensitive lines (Fig. 5G). Cytokine receptors and TAK1 were expressed to similar levels in both groups (Figure S5E, F). Most importantly, only sensitive GSCs secreted TNFα protein into the supernatant (Fig. 5F), which was required for the response to TAKi as pre-treatment of sensitive GSCs with Etanercept completely mitigated the growth defect caused by HS-276, as did inhibitors of RIPK1 and Caspases (Figs. 5H and S5G).

Collectively, these data demonstrate that GSCs sensitive to TAK1 inhibition are characterized by constitutive activation of inflammatory Interferon and NF-κB pathways. Our data suggest that these pathways are driven by autocrine ligand expression, and specifically secretion of TNFα renders cells vulnerable to induction of apoptosis by TAK1 inhibition.

### High immune signaling is a potential biomarker to identify GSCs responsive to TAK inhibition

After having identified activated immune signaling pathways as a key feature of GSCs vulnerable to TAK1 inhibition we next investigated the predictive nature of these signatures on an independent panel of 45 patient-derived GSCs cultures (Glioma Cellular Genetics Resource, GCGR). We used the 513 genes highly expressed in sensitive GSCs as a gene set (‘sensitivity signature’) as well as the curated IFNα, IFNγ, and TNFα response Hallmark sets and performed Gene Set Variation Analysis (GSVA) to determine the strength of each gene signature expression over the range of GSC samples.

There was a strong concordance of the GSVA scores derived from the different signatures in the same samples indicating our sensitivity signature can successfully identify cell lines with high activation of immune signaling pathways in an independent dataset (Fig. 5I). In accordance with the initial GSC dataset, we observed a striking enrichment of GSCs belonging to the mesenchymal GBM subtype in the high immune signaling pathway group (Fig. 5I) but no association with specific genetic alterations (Figure S6A).

As a next step, we ranked GSC based on the sensitivity signature score and tested their response to treatment with HS-276 with the prediction of a positive score indicating sensitivity and a negative score resistance to the drug. Remarkably, in this fresh GSCs panel, for all 14 tested lines, the sensitivity signature score accurately predicted response to HS-276 (Fig. 5J) and we confirmed the mechanism of RIPK1-mediated apoptosis via secretion of TNFα in two sensitive GSCs (Figure S6B). The predictive value of the sensitivity signature was not limited to GSCs but also correlated with *MAP3K7* gene dependency in 59 DepMap glioma (Fig. 5K) and non-glioma cancer cell lines (Figure S6C). As some of the strongest differentially expressed genes between sensitive and insensitive GSCs were ISGs (Fig. 5E), we tested if they could be used as a surrogate biomarker. Expression of ISGs *MX1*, *XAF1*, or *OAS2* strongly correlated with *MAP3K7* dependency in DepMap glioma lines (Figure S6D) and this correlation was still present, but weaker, in 930 non-glioma cell lines (Figure S6E).

In summary, we have identified a sensitivity gene expression signature, which can be used to successfully identify cancers with highly activated immune signaling, as well as to accurately predict the sensitivity of GSCs to TAK1 inhibitor HS-276. This sensitivity signature or ISG expression as a surrogate biomarker, might be used for selection of patients most likely to benefit from TAK1 targeted therapy.

### IFNγ pathway activation is required for TNFα mediated sensitization to TAK1 inhibition

**Fig. 6:**
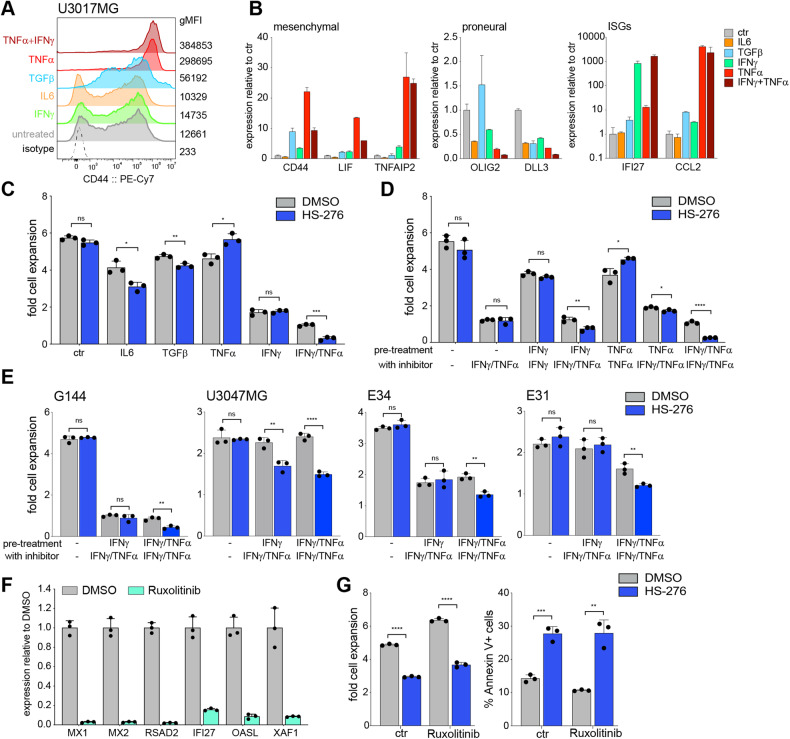
Combined IFNg and TNFa pathway activation is required to sensitize GSCs to TAK1 inhibition. **A** CD44 surface staining of U3017MG treated for 3 days with indicated cytokines. **B** qPCR of ISGs, mesenchymal, or proneural marker gene expression after cytokine treatment. Error bar indicates mean +/− SD of 2 technical replicates. **C**, **D** Fold cell expansion of U3017MG cells pre-treated for 3 days with indicated cytokines followed by 4 days of DMSO or HS-276. **E** Effect of HS-276 treatment on fold cell expansion of 4 proneural GSCs after pre-treatment with indicated cytokines. **F** qPCR of ISG expression in U3013MG cells after 48 h of Ruxolitinib treatment. **G** Fold cell expansion and %Annexin V positive cells in U3013MG cells pre-treated with Ruxolitinib followed by 4 days of HS-276.

Based on our findings of inflammatory and mesenchymal pathway activation as well as a clear causative role of the TNFα pathway in sensitive GSCs, we wondered if a transition to the mesenchymal subtype or mimicking an immune-activated state could induce dependency on TAK1 activity in insensitive lines. Different signaling pathways have been suggested to play an important role in the proneural-to-mesenchymal subtype transition of gliomas including cytokines IL6, TGFβ, and TNFα [37]. As expected, treatment of TAKi insensitive U3017MG cells with these cytokines led to the downregulation of proneural and concomitant upregulation of mesenchymal and inflammatory marker genes (Figs. 6A, B and S7A), with TNFα being the most potent inducer of the mesenchymal program, as has been previously reported [10]. Surprisingly, reprogramming with TNFα alone was not sufficient to sensitize U3017MG cells to TAK1 inhibition and pre-treatment with IL6 or TGFβ only showed minor effects. However, combined treatment with IFNγ and TNFα resulted in a strong sensitization effect (Fig. 6C), with similar results obtained in G144 cells (Figure S7B, C). Interestingly, IFNγ appears to be the key cytokine for the sensitization, as pre-treatment with IFNγ alone also sensitizes cells to TAK1 inhibition as long as TNFα is supplied together with HS-276 (Fig. 6D). However, in most proneural GSCs a combination pre-treatment with IFNγ and TNFα is required to induce TAK1 dependency (Fig. 6E). This sensitization completely depended on IFNγ pathway activation as IFNγ Receptor 1/2 or JAK1 knockout prevented cytokine-induced TAK1 dependency (Figure S7D). Intriguingly, two mesenchymal subtype GSCs insensitive to HS-276 as well as two untransformed fNSCs could not be reprogrammed with IFNγ/TNFα to acquire sensitivity to TAK1 inhibition (Figure S7E–H), even after exposure to both cytokines for 30 days (Figure S7I), suggesting cell intrinsic resistance to inflammatory cytokine reprograming in some GSCs and NSCs as well as immune-activation rather than a mesenchymal subtype being the key determining feature that leads to TAK1 dependency. As IFN pathway activation appears to be crucial in the process of acquiring TAK1 dependency, we wanted to test if this pathway was still required in TAK1-dependent GSCs. Pre-treatment of U3013MG cells with potent JAK1/JAK2 inhibitor Ruxolitinib resulted in shutdown of the IFN pathway indicated by strong downregulation of ISG expression (Fig. 6F), but sensitivity to HS-276 remained unchanged (Fig. 6G).

Taken together these results show that activation of inflammatory pathways with IFNγ and TNFα can induce TAK1 dependency in proneural GSCs, but not in untransformed NSCs, and pre-treatment with IFNγ is necessary for this reprogramming. In contrast, sensitive GSCs, characterized by the presence of a pro-inflammatory transcription network, the IFN pathway activity downstream of signal transducers JAK1/2 is dispensable for the response to TAK inhibition.

### Immune signaling activation is a common feature of TAK1-dependent cancer cells across malignancies

**Fig. 7:**
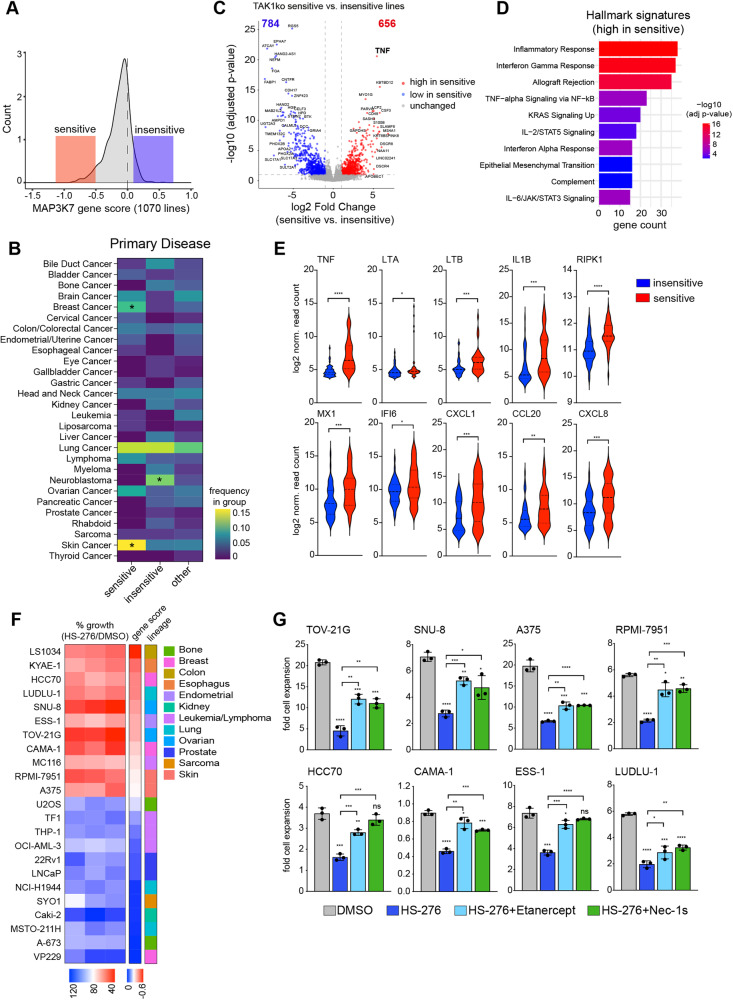
High immune signaling activation is a common feature of TAK1-dependent cancer cell lines. **A** Histogram of MAP3K7 dependency gene score from 1070 cancer cell lines. Highlighted are lines most sensitive (red) or insensitive (blue) to MAP3K7 depletion. **B** Heatmap of cell line frequencies in sensitive, insensitive, or other group plotted over different primary disease categories. * indicates significant enrichment in group with *p* < 0.05 (Fisher’s exact test). **C** Volcano plot of differentially expressed genes between sensitive (*n* = 47) and insensitive (*n* = 51) cancer lines. Significantly higher expressed genes in sensitive cell lines are colored in red, lower expressed genes in blue, and unchanged in gray. Dotted lines indicate cut-off value used to determine deregulated genes (absolute log2 Fold Change of >1 and adjusted *p*-value of <0.1). **D** Barplot of the 10 most significantly enriched Hallmark gene signatures highly expressed in lines sensitive to MAP3K7 depletion (gene set high, *n* = 656). **E** Violin plot of selected genes differentially expressed between cancer cell lines sensitive and insensitive to MAP3K7 depletion. **F** Heatmap of HS-276 effect on cell growth in 23 cancer cell lines with DepMap gene dependency score and lineage information. **G** Barplot of fold cell expansion in 8 TAKi sensitive cell lines with Etanercept and Nec-1s cotreatment.

Encouraged by the observation that the sensitivity signature defined in GCSs correlated with TAK1 dependency in other cancer cell lines (Figure S6C), we wanted to investigate the key features of all TAK1-dependent cancer lines independent of tissue origin. For this purpose, we stratified all 1070 DepMap cell lines with available *MAP3K7* knockout data in two groups as being the 5% most sensitive (gene score < -0.504) or the 5% most insensitive (gene score > 0.105) lines towards *MAP3K7* depletion, respectively (Fig. 7A). When exploring the primary disease type, we found skin and breast cancer cell lines were significantly enriched in the sensitive group, whereas neuroblastoma lines were strongly overrepresented in the insensitive group (Fig. 7B). This indicates that certain tissue lineages are more prone to display selective dependency on TAK1 function, but the tissue identity alone is not the main contributor as TAK1-dependent cell lines are distributed over many different cancer types. Next, we performed differential gene expression analysis, comparing the two groups to identify specific genes and pathways activated in cell lines dependent on TAK1. Strikingly, one of the most differentially expressed genes in the sensitive group was TNFR ligand TNFα (Fig. 7C). Moreover, the top hallmark signatures enriched in genes highly expressed in sensitive cancer lines were identical with the ones found in TAKi sensitive GSCs (Fig. 5D), including Interferon Alpha/Gamma Response, TNFα signaling via NF-κB, and Epithelial Mesenchymal Transition (Fig. 7D). Furthermore, all three TNFR ligands as well as several ISGs and RIPK1 were significantly overexpressed in sensitive lines (Fig. 7E). Treatment of a panel of selected DepMap cancer lines from diverse tissue origin with HS-276 showed high concordance of *MAP3K7* gene score with response to TAK1 kinase inhibition (Fig. 7F) and blockage of TNFα with Etanercept or inhibition of RIPK1 with Nec-1s could rescue the growth defect caused by TAK1 inhibition in eight tested sensitive cancer lines (Fig. 7G) indicating a conserved mechanism of dependency on TAK1 function in many cancer types.

This independent and unbiased analysis shows that constitutive immune activation, including Interferon and NF-κB signaling together with a mesenchymal transcription program, is a common feature of cancer cells dependent on TAK1 function and therefore our mechanistic findings of a selective, targetable vulnerability in immune-activated glioma stem cells can be extended to many different cancer types.

## Discussion

Identifying new therapeutic targets that are not derived from genome sequencing alone is a major unresolved issue in cancer. Here, using CRISPR genetic screens we have revealed a selective dependency on TAK1 kinase activity to suppress cell death in GSCs and cancer cells of diverse tissue origin with immune-activation and mesenchymal subtype features.

Mechanistically, we have shown that apoptosis triggered by TAK1 inhibition is exclusively dependent on RIPK1 kinase function and likely mediated via an inhibitory phosphorylation of serine 320 on RIPK1 (directly or indirectly) via TAK1. Our findings and the reported regulation of RIPK1 activity suggest a model in which TAK1 exerts its pro-survival role in immune signaling activated GSCs as a negative regulator of RIPK1 activity rather than through activation of a pro-survival NF-κB transcriptional program as has been suggested previously in GSCs and other cancers [38, 39]. This model is supported by our results showing that depletion of complex members Caspase-8 and FADD, in addition to RIPK1 itself, completely reverses the dependency on TAK1 function and the requirement of TAK1 kinase activity for cell survival.

We have shown and validated that high expression of ISGs is a key feature identifying cancer cells sensitive to TAK inhibition. Interferons play an important immunomodulatory role in several cancer types as they can promote tumor cell immune-evasion via downregulation of tumor-associated antigen presentation [40], upregulation of cytotoxic T cell inhibitor PD-L1 [41, 42], and decreasing sensitivity to NK cells via upregulation of MHC I class molecules [43]. IFNα/β can also activate the NF-κB pathway as well as TNFα expression, inducing cell survival and protecting tumor cells against apoptotic stimuli in a variety of cancer types [44–46]. Conversely, several IFN-stimulated genes including IFNβ have NF-κB response elements in their promoters, and NF-κB transcription factor RELA activity is required to maintain basal expression level of these genes prior to infection [47]. As both pathways are strongly interconnected, we expected that IFN and NF-κB transcriptional signatures would co-occur, indicating an overall immune signaling activated cellular state. Interestingly, our reprogramming experiments show that IFN pathway activation plays a key role in sensitizing proneural GSCs to TAK1 inhibition in the presence of TNFα but is dispensable once cells have acquired intrinsic immune-activation features and TAK1 dependency. However, more work is required to determine how these two pathways cooperate to induce dependency on TAK1 function.

The tumor microenvironment (TME) in GBM is a major source of IFN and TNF ligand production and has been shown to contribute to therapy resistance as well as acquisition of a more aggressive phenotype with mesenchymal features [48]. The transcriptional classified mesenchymal subtype of GBMs has been shown to display a greater frequency of infiltrating macrophages and microglia compared with proneural or classical tumors [6, 10]. Tumor-associated macrophages in glioma have been proposed to stimulate the proneural-to-mesenchymal transition through TNFα mediated NF-κB pathway activation [10]. Moreover, macrophage-derived IFNγ can stimulate transcriptional changes through epigenetic reprogramming in GSCs to promote immune evasion and this transcriptional state is maintained ex vivo [9]. These observations support our findings and suggest that the GSCs with high immune signaling signatures have experienced epigenetic changes in vivo imposed by a myeloid-enriched TME, leading to the sustained expression of the immune signature. Here we provide evidence that mimicking an inflammatory TME in vitro by treating GSCs with IFNγ and TNFα can induce TAK1 dependency, which untransformed NSCs appear to be protected from.

Overall, our findings suggest that the acquired immune evasion creates a new vulnerability in GSCs and other cancer cells, as they have gained dependency on TAK1 kinase activity. This Achilles heel can be exploited therapeutically by inhibiting TAK1 kinase activity as we have demonstrated in this study. Thus, a transcriptional state resulting from sustained immune attack has created a novel signaling context which can be therapeutically exploited to drive cell death. Importantly, we show that the sensitivity signature derived from genes highly expressed in TAK1-dependent cells can be used to predict sensitivity to TAK inhibition in cancer cells and might be used in the future to select patients benefitting from TAK1 targeted therapy.

Further studies are required to assess the therapeutic effect of TAK1 inhibition in GBM and other cancer models in vivo alone or in combination with other cytotoxic or immune-modulatory agents to target the main bulk of proliferating tumor cells and potentiate the treatment effects on overall tumor cell viability and growth. Moreover, given that the intratumoral heterogeneity and underpinning mechanisms of plasticity remain poorly defined, it will be important to determine how easily mesenchymal cells can adapt and resist TAK1 inhibition, and if a pro-inflammatory TME or even cancer immunotherapy can potentiate treatment efficacy. Although HS-276 has been shown to attenuate clinical symptoms in a mouse model for Rheumatoid arthritis, an inflammatory disease driven by TNFα mediated NF-κB stimulation via TAK1 [32], we have so far not been able to observe a survival benefit of treating mice transplanted orthotopically with mesenchymal gliomas with HS-276. Additional studies are required to understand this result, which could be due to low penetrations of HS-276 through the blood-brain barrier, effects of TAK1 inhibition on cells in the TME restricting tumor growth or the stability and potency of the compound in inhibiting TAK1 in this tumor context.

## Materials and methods

### Cell culture

HEK293FT, HCT116, U2OS, BJ hTERT, RPE-1 hTERT, LS1034, KYAE-1, HCC70, LUDLU-1, SNU-8, ESS-1, TOV21G, CAMA-1, MC116, RPMI-7951, A365, TF1, THP-1, OCI-AML-3, 22Rv1, LNCaP, NCI-H1944, SYO-1, Caki-2, MSTO-211H, A673, and VP229 were obtained from ATCC, DSMZ or academic labs and cultured according to the specifications of the providers in medium supplemented with 10% or 20% heat-inactivated FBS (HyClone), 1x penicillin/streptomycin (Thermo Fisher) and 2 mM L-glutamine.

U3013MG, U3031MG, U3024MG, U3017MG, U3047MG, U3065MG, and U3086MG GSCs were obtained from the human glioblastoma cell culture resource (HGCC) [14] and G166, G26, G7, G14, and G144 GSCs and human fetal neural stem cells U5, NS17ST-A, and NS9FB-B were provided by Steven Pollard and were cultured adherently in serum-free neural stem cell medium supplemented with N2, B27, and 10 ng/ml EGF and FGF-2 on PDL and Laminin I (Cultrex, R&D) precoated dishes as described previously [15]. GSCs from the Glioma Cellular Genetics Resource (GCGR) were grown similarly on PDL and Laminin I precoated dishes in serum-free DMEM/F12 medium (Sigma), supplemented with 4.5 g/L glucose, L-glutamine, 15 mM HEPES, 55 mg/L sodium pyruvate, non-essential amino acids, 0.012% BSA, penicillin/streptomycin, 100 μM 2-Mercaptoethanol, 0.5x N2/B27, 2 μg/ml Laminin as well as 10 ng/ml EGF and FGF-2. Detachment from plates and passaging of GSCs was performed with Accutase (Sigma). All cells used in the study were regularly confirmed to be mycoplasma negative by an in-house PCR-based method and were grown under standard tissue culture conditions at 37 °C with 5% CO_2_.

### Generation of stable and inducible Cas9 and dTAG-TAK1 cells

For the establishment of stably Cas9 expressing GSCs, HCT116, U2OS, BJ-hTERT, and RPE1-hTERT, cells were transduced with a lentivirus prepared from the lentiCas9-Blast construct (a kind gift from Feng Zhang, Addgene: 52962) and selected with 10 µg/ml blasticidin (Sigma). Doxycycline-inducible Cas9 GSCs were generated by delivering piggyBac plasmids (pB-tetO-Cas9-P2A-mCherry-hygro, pB-rTTA-zeo, PBase) via nucleofection (Amaxa, program A-033), followed by selection with hygromycin (150 µg/ml) for one week and three rounds of sorting on an Aria III cell sorter (BD). First round of sorting was performed 48 h after induction with 200 ng/ml doxycycline (dox) for high mCherry expressing cells, followed by a second sort for mCherry negative cells >7 days after sort recovery including dox withdrawal. The final iCas9 GSCs were obtained after subjecting the cells to another round of selection for mCherry positive cells after exposure to dox.

U3013MG dTAG-TAK1 degron cells were generated by overexpression of dTAG-TAK1 (pLEX305-2xHA-dTAG-MAP3K7) in U3013MG iCas9 sgMAP3K7_32 cells. Endogenous MAP3K7 knockout was performed by treatment with dox for 48 h. mCherry positive (Cas9) and BFP-positive (sgRNA) cells were single cell sorted in 96-well plates on an MA900 sorter (Sony) and clones were screened for the absence of endogenous TAK1 by PCR and western blot as well as exogenous dTAG-TAK1 by western blot with an antibody against the HA-tag.

### Treatment with cytokines and chemicals

Development and synthesis of small-molecule TAK1 kinase inhibitors Takinib and HS-276 was described previously [31, 32]. TAK1 inhibitors were solubilized in DMSO as 10 mM stocks and treatment of GSCs was performed with indicated concentrations for the duration of the experiment. Inhibitor in the medium was refreshed at each cell passage for cumulative growth assays.

For rescue experiments, cells were pre-treated for 1 h with 10 μM RIPK1 kinase inhibitor Necrostatin-2 (Nec-1s, Cayman Chemicals), 10 μg/ml Etanercept (Enbrel, Immunex Corp.) or 20 μM zVad-fmk (UBPBio), before addition of TAK1 inhibitor HS-276 or Takinib. Pan-caspase inhibitor zVad-fmk was added to sgMAP3K7 expressing iCas9 cells 48 h after induction of knockout with doxycycline at a concentration of 20 μM until analysis of cell death markers 4 days after knockout induction. For TAK1 protein degradation experiments, dTAG-TAK1 cells were treated with 100 nM dTAG^V^-1 ligand (Tocris) for indicated durations. Doxycycline (Sigma) was used at 200 ng/ml in all experiments. Etanercept (Enbrel, Immunex Corp.) was obtained via the Sloan Kettering Institute drug requisition program, reconstituted as 25 mg/ml in sterile water and used at indicated concentrations to treat cells. Cytokines (Peprotech) were added at 10 ng/ml for 3 days before analysis of marker expression or treatment with HS-276. Cytokine treatment was continued during 4 days treatment with HS-276. JAK1/2 inhibitor Ruxolitinib (Tocris) was added to the cells at 500 nM for 48 h prior to treatment with HS-276 or analysis of ISG expression by qPCR. GSCs were treated with 250–1000 nM p38α/β inhibitor LY2228820 (Selleckchem) for 4 days.

### Molecular cloning and plasmids

The pLV-U6-sgRNA-SFFV-puro-P2A-EGFP vector was generated by substituting Cas9 open reading frame with a puromycin resistance cassette in the pL-CRISPR.SFFV.GFP plasmid (Addgene, 57827) [49]. Individual sgRNAs were designed using designer tool CHOPCHOP (https://chopchop.cbu.uib.no/) with prioritization of cutting within functional protein domains, lowest off-target and highest on-target prediction score. For sgRNA cloning into pU6-sgRNA-EF1α-puro-T2A-BFP (Addgene, 60955), oligos were annealed in annealing buffer (100 mM NaCl, 10 mM Tris-Hcl pH 7.4) and ligated into BstXI+BlpI (NEB) digested pU6-sgRNA-EF1α-puro-T2A-BFP. For sgRNA cloning into pLV-U6-sgRNA-SFFV-puro-P2A-EGFP, the oligos were phosphorylated by T4 PNK (NEB) and annealed in the T4 ligation buffer (NEB). The oligos and plasmid mixture was then subjected to digestion by BsmBI (NEB) and ligation by T4 ligase (NEB) (4 cycles of 42 °C—5 min and 16 °C—5 min, inactivation 55 °C—15 min). Correct insertion of sgRNA sequence was confirmed by Sanger Sequencing (Genewiz). All sequences of individually cloned sgRNAs can be found in Table S2.

Gateway compatible entry vector containing human MAP3K7 was obtained from Addgene (23693, pDONR223-MAP3K7). Silent mutations in the PAM site and seed regions of sequences matching sgMAP3K7_15 and sgMAP3K7_32 as well as amino-acid substitution mutation K63W were introduced by site-directed mutagenesis. For localization mutants, NES from human NPM1 gene or SV40 3xNLS were added to the N-terminus of MAP3K7. Gateway cloning using LR Clonase II enzyme mix (Invitrogen) was used to generate lentiviral expression vectors of MAP3K7 with a C-terminal V5 epitope tag (pLEX_306, Addgene, 41391). dTAG-TAK1 overexpression construct was generated by cloning PAM mutant MAP3K7 into pLEX_305-N-dTAG (Addgene, 91797).

### Lentivirus production and transduction

HEK293FT cells were transfected with lentiviral construct of interest together with packaging plasmids psPAX2 (Addgene, 12260) and pMD2.G (Addgene, 12259) using 3 μg linear polyethylenimine (Polysciences) per 1 μg total plasmid DNA. The day after transfection, HEK293FT cells were rinsed in PBS and medium was changed to the culture medium of the cell line intended to be transduced. Viral supernatant was harvested 72 h after transfection, passed through a 0.45 μm filter unit and immediately used to transduce recipient cells or aliquoted and stored at −80 °C. Transduction was performed by adding viral supernatant to adherent cells and incubating overnight. For generating stable cell lines expressing sgRNAs, MAP3K7, or Cas9, antibiotic selection was started 24 h after transduction. For rescuing dTAG-TAK1 or HS-276 phenotypes, U3013MG iCas9 cells were transduced with sgRNA expressing virus, selected with 1 μg/ml puromycin and knockout was induced with dox treatment 7 days prior to TAK1 degradation with dTAG^V^-1 or inhibition with HS-276.

### Competitive/cumulative growth and cell viability assays

Cell expressing constitutive or doxycycline-inducible Cas9 were transduced with viral supernatant from respective pU6-sgRNA-EF1α-puro-T2A-BFP constructs with an MOI between 0.3 and 0.7. Three days after transduction, percentage BFP positivity was measured as a reference point by flow cytometry and cells were reseeded in three biological replicates. For competitive growth assays with constitutive Cas9, BFP positivity was assessed by flow cytometry at each passage in individual wells and subsequently reseeded. BFP percentage was normalized to the reference time point after transduction. For competitive growth assays in GSCs with doxycycline inducible Cas9 (iCas9), 3 days after transduction with sgRNA virus and assessment of BFP percentage, four replicate wells were seeded with three being treated with 200 ng/ml doxycycline to induce Cas9 expression and one well being kept uninduced and BFP percentage in each well was measured by flow cytometry at each passage. At each time point BFP percentage was normalized to the uninduced control condition. For testing the requirement of apoptosis mediators on the TAK1 knockout phenotype, U3013MG iCas9 cells were mixed with U3013MG iCas9 sgMAP3K7_32-BFP cells in a 1:1 ratio and transduced with pLV-U6-sgRNA-SFFV-puro-P2A-EGFP virus targeting the gene of interest. Competitive growth assay was performed as described above with one replicate being uninduced and three replicates treated with doxycycline. Percentage of BFP-positive cells was assessed in the subpopulation of GFP expressing cells (second sgRNA) and normalized to uninduced (no dox) control well at each time point. All data were acquired on a Cytoflex LX flow cytometer (Beckman Coulter) and analyzed with FlowJo v10.8 (BD). For cumulative growth curves, 100,000 GSCs were seeded in laminin-precoated 12-well plates in triplicates and treated with 200 ng/ml doxycycline for knockout studies or 3 μM HS-276 for TAK1 inhibitor studies. Every 4 days, cells were harvested, counted manually using a Burker counting chamber (Sigma) and 100,000 GSCs were reseeded for each condition and replicate.

Cell viability assays were performed by seeding 5–10,000 GSCs/well into 96-well plates, treating with indicated drugs in triplicates for 5–6 days and assessing cell viability by addition of Cell Titer Blue reagent (Promega) according to the manufacturing’s protocol. Fluorescence intensity was measured on a Biotek plate reader and data were normalized to DMSO treatment conditions after subtraction of wells containing culture media only.

### Annexin V/Caspase staining and cell cycle analysis

150,000 glioma stem cells per replicate were harvested with Accutase (Sigma), washed in Annexin V binding buffer (BD), and incubated for 30 min with APC-Annexin V dye (BD). After two more washing steps, stained cells were resuspended in Annexin V binding buffer containing 10 ng/ml DAPI. Data were acquired on a Cytoflex LX flow cytometer (Beckman Coulter) and analyzed with FlowJo v10.8 (BD). Apoptotic cells were determined by first excluding debris and subsequent gating on APC positive/DAPI negative cells (early apoptotic) and APC positive/DAPI positive (late apoptotic) cells. For graphs showing overall Annexin V positive cell fraction, overall percentage APC positive cells were quantified after exclusion of debris. To determine Caspase activity in GSCs after treatment, 100,000 GSCs per biological replicate were harvested with Accutase, transferred to a 96-well U-shaped plate, resuspended in 100 µl medium containing 0.3 µl FITC-VAD-FMK substrate (CaspGLOW kit, Biovision) and incubated for 1 h at 37 °C in a tissue culture incubator with 5% CO_2_. Cells were subsequently washed twice in wash buffer (CaspGLOW kit, Biovision) and resuspended in 150 μl wash buffer containing 10 ng/ml DAPI before acquisition on a Cytoflex LX flow cytometer. For Cell Cycle Analysis, 4 days after knockout induction, GSCs were labeled for 1 h with 10 µM EdU and stained with Click-it Plus EdU Flow Cytometry Assay kit (Invitrogen) according to manufacturer’s instructions. 1 µg/ml DAPI was added to the samples before acquisition on a Cytoflex LX flow cytometer.

### Surface staining by flow cytometry

100,000 GSCs were harvested with Accutase (Sigma), washed in PBA (0.5% BSA in PBS), and incubated for 30 min with fluorescently conjugated antibodies against CD44 (eBioscience), DR5, FAS, or matching isotype controls (Biolegend) in the dark on ice. After two washing steps with PBA, stained cells were resuspended in 10 ng/ml DAPI containing PBA. Data were acquired on a Cytoflex LX flow cytometer (Beckman Coulter) and analyzed with FlowJo v10.8 (BD).

### TNFα ELISA

Equal number of GSCs were seeded in 12-well plates and conditioned supernatant was collected from each well after 7 days of incubation. Cellular debris was removed by centrifugation for 4 min at 500 × *g* and 100 µl of cleared supernatant was used for cytokine measurement. Concentration of soluble TNFα was determined using TNF-alpha DuoSet ELISA kit (R&D) according to manufacturing’s protocol instructions. Each biological replicate was measured twice.

### Immunofluorescent staining

U3013MG cells stably expressing C-terminally V5-tagged wild-type TAK1, 3xNLS::TAK1, or NES::TAK1 were seeded on PDL/laminin precoated glass cover slips and were stained two days after plating as follows: fixed in 4% PFA for 15 min, permeabilized and blocked with staining buffer (0.2% Triton X-100 and 2% goat serum in PBS) for 1 h, incubated with primary antibody in staining buffer overnight at 4 °C, next day washed three times in wash buffer (0.2% Triton X-100 in PBS), stained with secondary antibody in staining buffer for 1 h, washed three times in wash buffer followed by 5 min incubation with wash buffer containing 10 ng/ml DAPI and mounting on a glass slide with Vectashield (Vector). Images were acquired on an Eclipse Ti Inverted fluorescent microscope (Nikon) and processed using Fiji software (ImageJ).

### Cellular fractionation

GSCs were washed twice in PBS followed by a wash in 250 µl Buffer A (10 mM Tris-HCl pH 7.9, 10 mM KCl, 1.5 mM MgCl_2_). Cytoplasmatic fraction was extracted by resuspending cells in 150 µl of Buffer A complemented with 0.2% NP40, centrifugation at 3300 × *g* for 15 min and harvesting the supernatant. Pelleted material containing the nucleus was washed twice in PBS, resuspended in 80µl Buffer C (20 mM Tris-HCl pH 7.9, 420 mM NaCl, 2 mM MgCl_2_, 0.2 mM EDTA, 0.5 mM DTT, 20% Glycerol, 0.1% NP40) and incubated on ice for 60 min. The nucleoplasmic fraction was collected from the supernatant after centrifugation at 20,000 × *g* for 30 min. The insoluble chromatin fraction was washed twice with Buffer C and resuspending in extraction buffer (300 mM NaCl, 50 mM Tris, 1% SDS, 0.5% Triton X-100, 1 mM DTT) containing 50 units/ml Benzonase (Sigma) by incubation for 30 min at 37 °C on a shaker. Protein concentration was quantified by Bradford assay (Biorad) and equal amount of protein per fraction was loaded on a Tris-glycine SDS-PAGE gel (Invitrogen). All buffers were supplemented with Halt Protease Inhibitor Cocktail (Thermo Fisher), samples were kept on ice during the entire procedure and all centrifugation steps were performed at 4 °C.

### Complex IIb formation

GSCs in 10 cm dishes were treated with DMSO + 5 µM Emricasan or 10 ng/µl TNFα + 3 µM HS-276 + 5 µM Emricasan for 2 or 4 h prior to lysis with IP buffer (30 mM Tris, 120 mM NaCl, 2 mM EDTA, 2 mM KCl, 1% Triton X-100, 10% Glycerol). Immunoprecipitation with 5 µl anti-Caspase 8 antibody was performed on pre-cleared lysates overnight at 4 °C with protein A/G agarose beads followed by 4 washes with IP buffer prior to elution with Laemmli buffer and Western blotting.

### Western blot analysis

Total protein from cell pellets was isolated by lysis in extraction buffer (300 mM NaCl, 50 mM Tris, 1% SDS, 0.5% Triton X-100, 1 mM DTT) containing 35 units/ml Benzonase (Sigma) for 10 min at room temperature. Concentration of proteins was determined by Bradford assay (Biorad) with a y-globulin reference standard curve. Laemmli buffer containing beta-mercaptoethanol (Fisher) was added to the lysates to a final concentration of 1x and samples were heated to 90 °C for 10 min before loading 50-60ug total protein per lane on a Tris-glycine SDS-PAGE gel (Invitrogen). Proteins were transferred onto a nitrocellulose membrane (LI-COR), followed by membrane blocking with 5% milk for 1 h and incubation with primary antibody overnight at 4 °C. After washing and incubation with IRDye secondary antibodies (LI-COR), membranes were imaged on an Odyssey CLx System (LI-COR) and images were processed with ImageStudio Lite software (LI-COR). Table S12 contains detailed information of all antibodies used. Images of uncropped Western blots are provided as supplementary information file.

### Design of domain-focused human sgRNA library

A list of 1387 human proteins involved in epigenetic regulation was compiled from the literature and was used to generate an epigenome-wide domain-focused CRISPR/Cas9 sgRNA library. The hg19 PFAM domain annotations was used to identify any functional domain in these proteins, and 5 to 10 sgRNAs targeting within this domain were selected based on published design strategies [49, 50]. An oligo pool consisting of 12,472 60-bp oligos targeting these 1387 epigenetic regulators, as well as 175 positive and 1010 negative non-targeting controls, was synthesized by CustomArray. The library was amplified by polymerase chain reaction, cloned into the pLV-U6-sgRNA-SFFV-puro-P2A-EGFP lentiviral expression vector, amplified and sequence verified by next-generation sequencing. A detailed cloning and library amplification procedure has been published previously [49]. Table S1 contains sequences of all the sgRNAs in the domain-focused CRISPR library.

### CRISPR/Cas9 knockout screen and analysis

The domain-focused epigenetic sgRNA library was used in lentiviral pooled format to transduce Cas9-BSD expressing GSCs at ∼500-fold representation of the library at an MOI of 0.3. Two days after transduction, 1–2 μg/ml puromycin was added for 5 days. A portion of cells were harvested as day 0 time point after selection was completed. The rest of the cells were then passaged to maintain 500-fold representation and cultured for an additional 35–38 days (eight to ten cell doublings). During the screening period antibiotic selection for Cas9 and sgRNA expression was maintained at 2.5 μg/ml blasticidine and 0.5 μg/ml puromycin, respectively. Each screen was performed in two biological replicates. Genomic DNA was extracted using DNeasy Blood & Tissue kit (Qiagen), and a two-step PCR procedure was employed to amplify sgRNA sequences and to incorporate deep sequencing primer sites onto sgRNA amplicons. Purified PCR products were quantified by Qubit dsDNA HS Assay Kit (Thermo Fisher) and KAPA Library Quantification Kit (Roche), pooled in equimolar quantities and sequenced on a NextSeq 550 instrument using a single-end 75 bp kit (Illumina). Reads were mapped against the sgRNA library with bowtie [51] (v1.2.2) with parameter -m 1 -v 1. Differential abundance of sgRNAs between d35/38 and d0 was performed in R (v3.6.1) using DESeq2 [52] (v 1.26.0) and genes were considered a hit with two independent sgRNAs meeting cut-off values of FDR-adjusted *p*-value < 0.05 and log2 fold change < −2. DESeq2 result tables are provided in Tables S4 and S5. Raw and mapped read count files are available at the Gene Expression Omnibus database (GSE208696).

### RNA-seq and analysis

Total RNA from all cell line and tissue samples was extracted using RNeasy Plus Mini Kit (Qiagen) according to the manufacturer’s protocol. GSC RNA concentration was determined on a Nanodrop (Thermo Fisher) and RNA quality was confirmed on a Bioanalyzer 2100 RNA 600 Nano Chip (Agilent) to have a RIN value of >9. Libraries for next-generation sequencing were prepared with 500 ng total RNA input using a TruSeq RNA Library preparation kit v2 (Illumina) according to manufacturer’s protocol. Individual library samples were quantified by Qubit dsDNA HS Assay Kit (Thermo Fisher) and Bioanalyzer 2100 HS DNA Chip (Agilent) and pooled in equimolar quantities. Pooled samples were sequenced with a single-end 75 bp read kit on the NextSeq 550 platform (Illumina). Reads were trimmed using Trimmomatic v0.36 and mapped to the human GRCh37.p13/hg19 genome using HISAT2 [53] (v2.2.1) with default parameters. Transcript count matrix was generated by using featureCounts [54] (subread package 2.0.1) with gencode.v19.annotation.gtf for transcript annotation. RNAseq read count data from CCLE lines (release 22Q1) was downloaded from the DepMap data portal. Differential gene expression analysis was performed in R (v3.6.1) using DESeq2 [52] (v1.26.0). Significantly differentially expressed genes (DEGs) between GSCs sensitive (*n* = 6) or insensitive (*n* = 6) to TAK1 inhibitor treatment or TAK1 knockout sensitive or insensitive DepMap cancer cell lines were defined with cut-off values FDR-adjusted *p*-value < 0.10 and absolute log2 fold change > 1. Differentially regulated transcripts are provided in Tables S6 and S10. For heatmap visualization of DEGs, samples were z-score normalized and plotted using ‘pheatmap’ package (v1.0.12) in R. Annotation of DEGs was performed using Enrichr [55] and gene set enrichment analysis was performed using a pre-ranked gene list based on the Wald statistics from the RNAseq DEG analysis with GSEA software (4.2.3) [56] against MSigDB release 7.5.1. Results are provided in Tables S7, S8 and S11. Raw and mapped read count files are available at the Gene Expression Omnibus database (GSE208697).

For GCGR-GSC transcriptional analysis, RNA samples were collected within first 10 passages after isolation from patient tumors, run on Bioanalyzer RNA 6000 Nano chips to determine RIN values and sample concentrations were measured using Qubit RNA BR assay (Thermo Fisher) and 200 ng of starting material was used. RNA-seq libraries were prepared using the KAPA mRNA Hyper prep kit with KAPA SeqCap Adapters (Roche). Each library was quantified using Qubit dsDNA HS assay and average fragment size was determined using Bioanalyzer DNA 1000 or DNA HS. Molarity of each library was calculated using the Qubit and BioA results and then normalized to ~10 nM and pooled, 24 libraries per pool. Each pool was then quantified using Qubit dsDNA HS assay and average fragment size determined using Bioanalyzer DNA HS. Pooled library molarity was determined using Qubit and BioA. The GCGR libraries were sequenced on HiSeq 2500 instrument (Illumina). Dilute and denature was done according to manufacturer’s instructions for “Dilute and Denature Libraries for cBot Clustering,” Standard normalization method. Each 24-library pool was run on two lanes of a HiSeq High Output flow cell. All GCGR-GSCs were processed at passage 3 from derivation. RNA sequencing reads from GCGR tumor and cell line samples were trimmed with TrimGalore (v0.5.0) and aligned to the hg38 human genome using the pseudo aligner Kallisto with default parameters (v0.44.0) [57]. Abundance estimates were imported via the R package Tximport (v1.8.0) [58] and subsequent normalization was completed via DESeq2 (v1.27.32) as regularized-logarithm transformation (rlog) [52].

### RT–qPCR

Total RNA was extracted using RNeasy Plus Mini Kit (Qiagen) in accordance with the manufacturer’s protocol. 500 ng of total RNA was subjected to reverse transcription using Transcriptor Universal cDNA Master (Roche). RT–qPCR reactions were set up in duplicates using PowerUp SYBR Green Matermix (Thermo Fisher) and run on a QuantStudio 6 Flex instrument (Thermo Fisher). Relative quantitation was performed by a delta Ct method with normalization to housekeeping gene *RPLP0*. All primer sequences are listed in Table S3.

### Gene set variation analysis

Cell line and tissue samples were processed independently. Signature scoring for each sample set was completed using glioblastoma transcriptional subtypes (mesenchymal, proneural, classical) [6] and hallmark signatures [59] using the R package gsva (v1.35.7) [60], with the gsva method, on rlog normalized reads obtained from normalization via DESeq2. All gene signatures used are listed in Table S9.

### Quantification and statistical analysis

Sample size descriptions are detailed in Figure legends. No data points were excluded from this study. All in vitro assays were performed in biological triplicates and error bars on barplots indicate mean + SD unless stated otherwise. The investigators were not blinded to data allocation during experiments and outcome assessment. Statistical analysis was performed using GraphPad Prism 9.2.0. For direct pairwise comparisons where appropriate, an unpaired two-tailed t-test was used unless otherwise stated and *p*-values are denoted as follows *p < 0.05, **p < 0.01, ***p < 0.001, ****p < 0.0001, ns > 0.05. Data distribution was assumed to be normal, but this was not formally tested. Pearson’s correlation analysis of MAP3K7 gene dependency score and gene expression or signatures was performed using R stats package v3.6.1. Log2 normalized gene expression values and CRISPR dependency scores were downloaded from the DepMap data portal (release 22Q1). Pearson’s correlation coefficient and *p*-values are displayed in the graph.

### Supplementary information

Supplementary Figures

Supplemental Information Western blots

Table S1

Table S2

Table S3

Table S4

Table S5

Table S6

Table S7

Table S8

Table S9

Table S10

Table S11

Table S12

## Data Availability

CRISPR/Cas9 dropout screen in two glioblastoma stem cell lines with a domain-focused sgRNA library targeting 1387 human proteins involved in epigenetic regulation has been deposited in the Gene Expression Omnibus (GEO) under accession number GSE208696. Baseline RNA-seq expression data from 12 glioblastoma stem cell lines has been deposited under GEO accession number GSE208697. Both datasets are publicly available in GEO at GSE208698. GCGR cell line RNA-seq datasets will be made available for download at http://gcgr.org.uk. This paper does not report original code. Any additional information required to reanalyze the data reported in this paper is available from the lead contact upon request.

## Materials availability

All reagents generated in this study (including cell lines and plasmids) are available upon request from KH.
