# Supplementary material for: TAK1 inhibition leads to RIPK1-dependent apoptosis in immune-activated cancers
Source: Cell Death Dis. 2024 Apr 17;15(4):273. doi: 10.1038/s41419-024-06654-1 (PMC11024179; doi:10.1038/s41419-024-06654-1)
Supplement: Supplementary file 1 — Supplementary Figures [file 41419_2024_6654_MOESM1_ESM.docx]

# Supplementary Figures

**­­­**

**
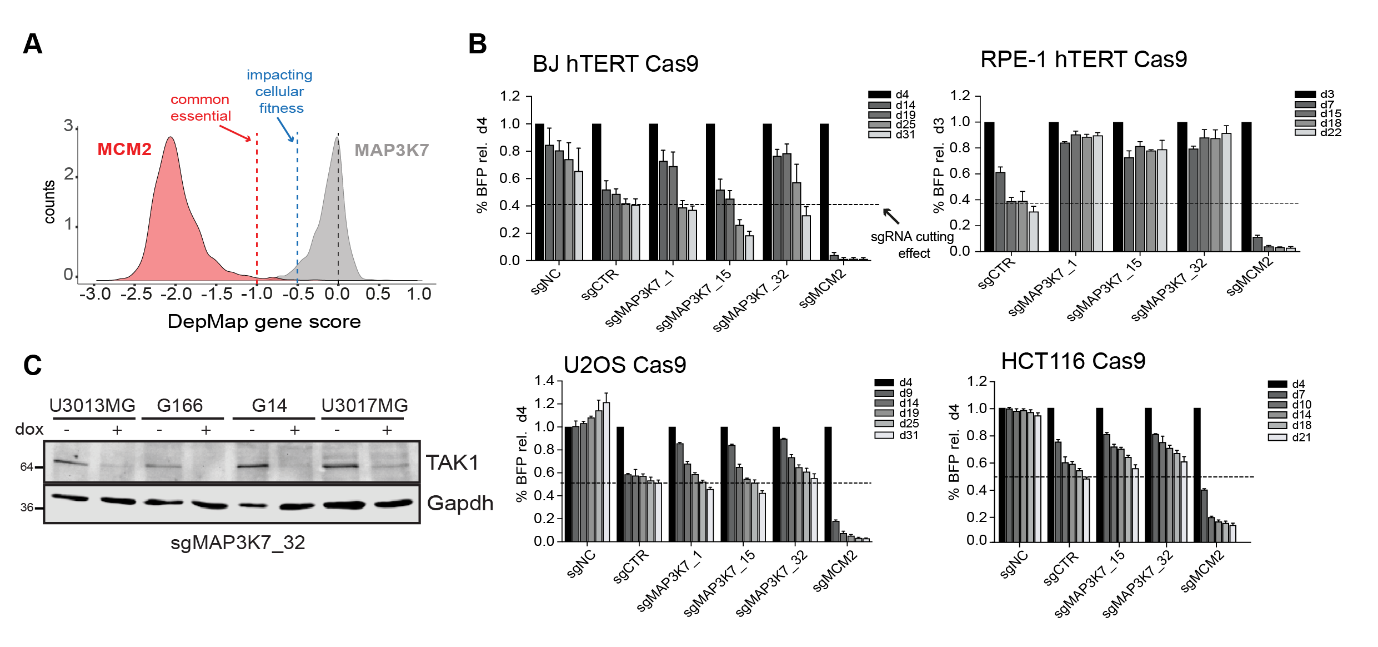
Figure S1: Transformed and untransformed cell lines of diverse tissue origin are insensitive to loss of TAK1 function. A**, Histogram of DepMap gene score of *MAP3K7* and *MCM2* (common essential gene) from 1070 cancer cell lines. **B**, Bar plot of competitive growth assay in Cas9 expressing BJ hTERT, RPE-1 hTERT, U2OS and HCT116 cells. Percentage of BFP positive cells in population was measured by flow cytometry and depicted relative to d3-4 measurement. sgNC (non-targeting control sgRNA), sgCTR (targeting control sgRNA cutting outside a coding gene), sgPRMT5/sgMCM2 (essential gene positive control sgRNAs). Error bar indicating mean +/- SD for 3 biological replicates at each time point. **C**, Western blot for TAK1 protein levels in iCas9 GSCs expressing sgMAP3K7_32 4 days after induction of Cas9 expression with doxycycline (dox) treatment.

**
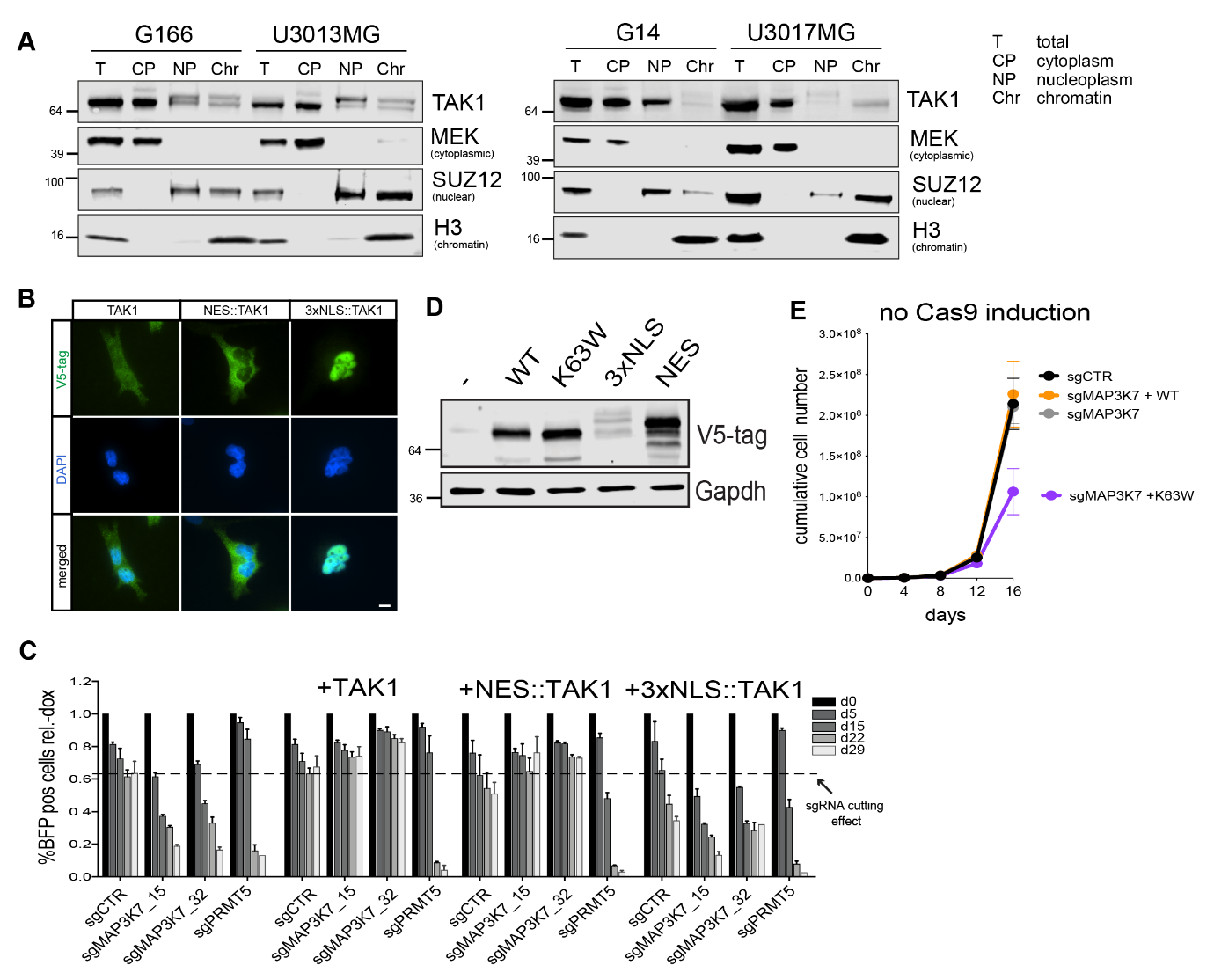
Figure S2: The cytoplasmic kinase activity of TAK1 is required for survival of GSCs. A**, Western blots of cellular fractions prepared from indicated GSCs. **B**, Immunofluorescent staining for the indicated V5-tagged TAK1 proteins. Scale bar = 10 µm. **C**, Competitive growth assay with complementation by overexpression of TAK1, NES::TAK1 or 3xNLS::TAK1. **D**, Western blot of TAK1-V5 constructs in GSCs. **E**, Cumulative growth assay in sgCTR and sgMAP3K7 cells without Cas9 induction overexpressing wild type TAK1, or catalytically inactive TAK1^K36W^ mutant. Error bars indicate mean + SD for 3 biological replicates at each time point.


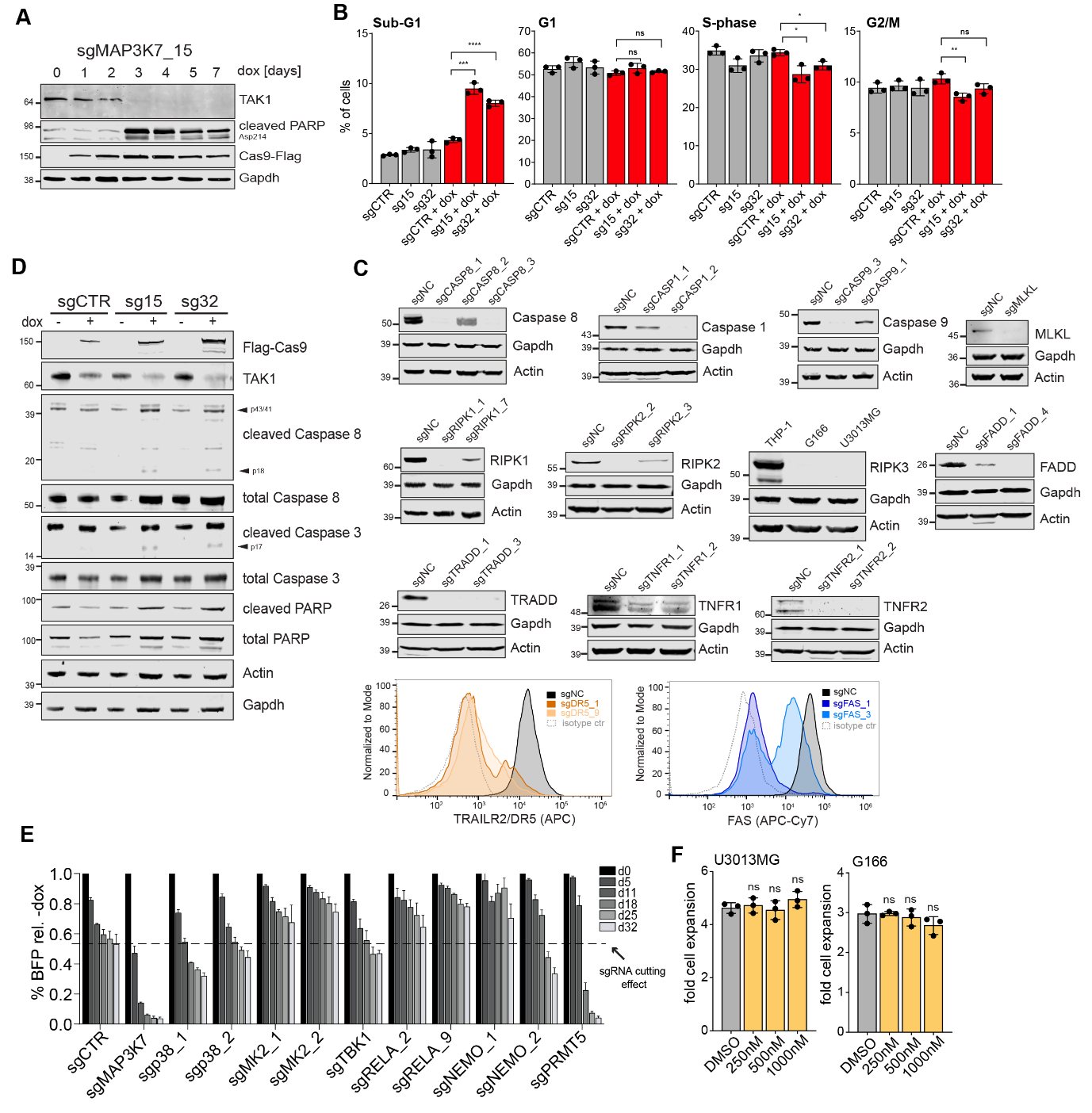
Figure S3. GSCs undergo apoptosis marked by PARP cleavage and Caspase activation upon TAK1 knockout. A, Representative western blot of time course experiment of sgMAP3K7_15 expressing U3013MG iCas9 cells upon induction of Cas9 by dox treatment for up to 7 days. B, Cell cycle phases determined by EdU incorporation and nuclear content staining with DAPI in U3013MG cells 4 days after induction of TAK1 knockout (sg15 and sg32, + dox). Error bars indicate mean + SD from 3 biological replicates. C, Western blots or cell surface stainings of indicated proteins in sgRNA expressing GSCs after 7 days of knockout induction with doxycycline. D, Western blots of different apoptosis markers 4 days after induction of Cas9 expression with dox in sgCTR, sgMAP3K7_15 and sgMAP3K7_32 expressing G166 cells. E, Competitive growth assay in U3013MG cells with knockout of different kinases and NF-κB signaling components. Error bars indicate mean + SD for 3 biological replicates at each time point. F, Fold cell expansion of U3013MG and G166 cells treated with DMSO or increasing concentrations of p38α/β kinase inhibitor LY2228820 for 4 days. ns = no significant difference relative to DMSO.

**
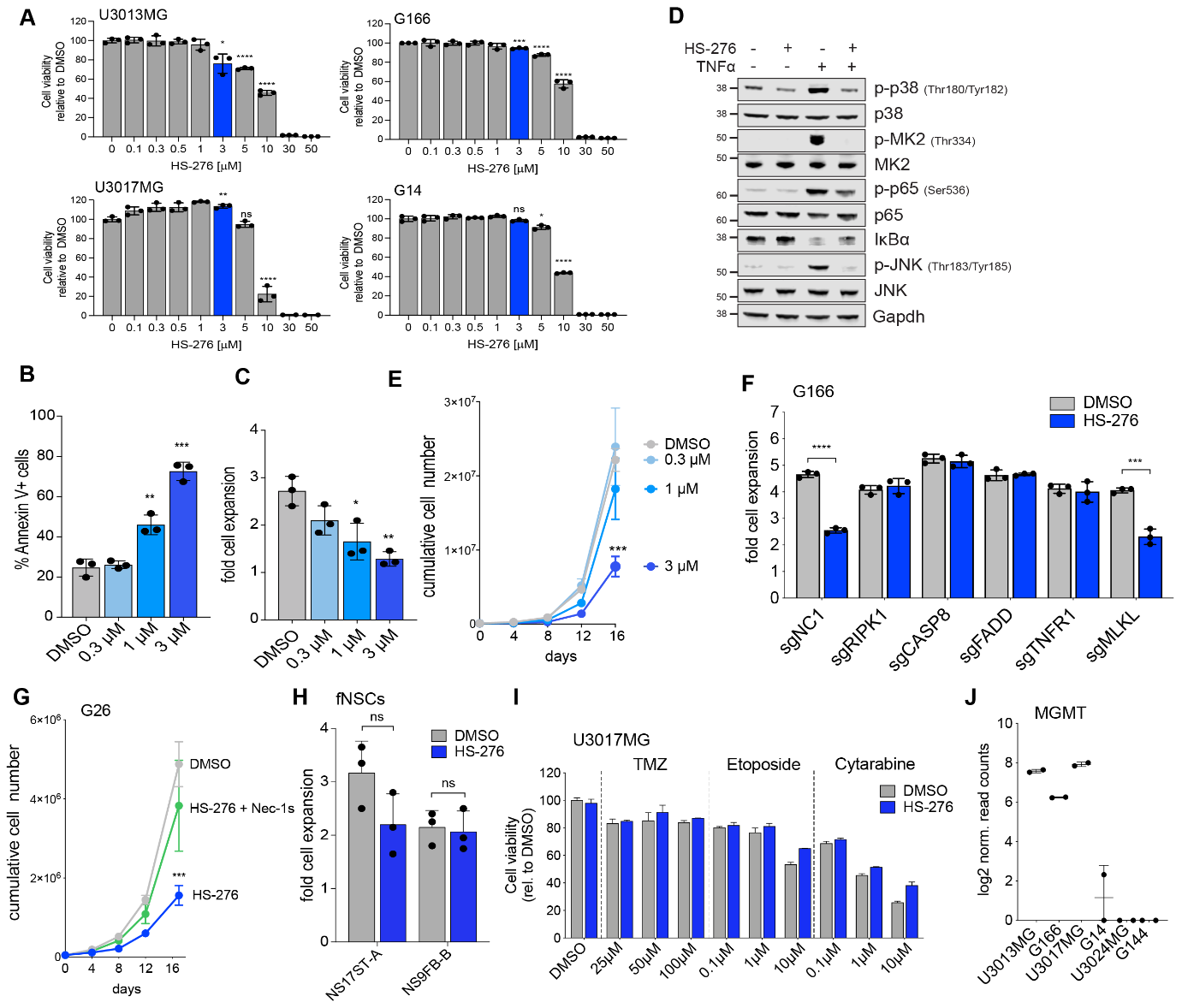
Figure S4**. **Novel TAK1 inhibitor HS-276 is selective for TAK1 dependent GSCs**. **A**, Barplot of cell viability normalized to DMSO control measured in four GSCs treated for 5 days with increasing concentrations of HS-276. **B-C**, Barplot of %Annexin V positive cells (B) and fold cell expansion (C) in U3013MG cells treated with DMSO or increasing concentrations of TAK1 kinase inhibitor HS-276 for 4 days. **D**, Western blot of U3013MG cells treated for 1 hour with 3µM HS-276 followed by 5 minutes of 10ng/ml TNFα. **E**, Cumulative growth assay over 16 days of U3013MG cells treated with indicated concentrations of HS-276. **F**, Fold cell expansion of G166 cells with knockout of TNFR pathway members upon 4 days treatment with DMSO or 3µM HS-276. **G**, Cumulative growth assay of G26 cells treated with DMSO, HS-276 or a combination of HS-276 and Nec-1s. **H**, Barplot of fold cell expansion within 4 days of treatment with DMSO or 3µM HS-276 in fetal neural stem cells NS17ST-A and NS9FB-B. **I**, Barplot of cell viability relative to DMSO in U3017MG cells treated with indicated chemotherapeutic drugs in increasing concentrations alone or in combination with HS-276 for 4 days. **J**, MGMT gene expression shown as log2 normalized read counts from 2 biological RNAseq replicates in indicated GSCs. All error bars indicate mean +/- SD of 3 biological replicates. p-values from t-test comparison with DMSO treatment are indicated with ****p<0.0001, ***p<0.001, **p<0.01, *p<0.05, ns = not significant

**
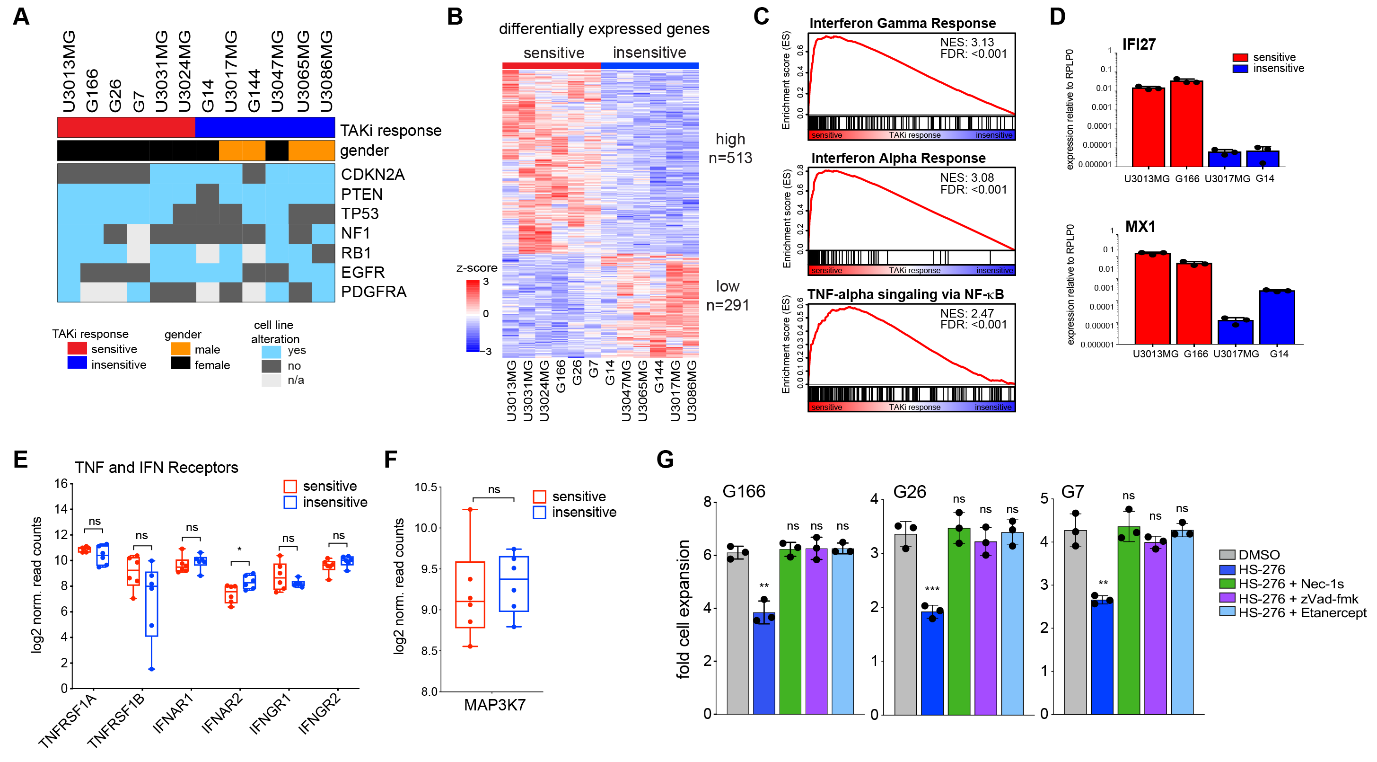
**

**Figure S5. TAK1 inhibitor sensitive GSCs are characterized by high immune-activation gene expression signatures**. **A,** Heat map of TAK1 response, gender and genetic alterations in GSC lines. n/a, data not available. **B,** Heat map of 804 differentially expressed genes identified in the HS-276 sensitive vs. insensitive GSCs. **C**, Gene set enrichment analysis (GSEA) plots of the Hallmark gene signatures of Interferon Gamma/Alpha Response and TNFα signaling via NF-κB. NES, normalized enrichment score. **D**, Barplot of gene expression of interferon-stimulated genes *IFI27* and *MX1* in sensitive and insensitive GSCs measured by qPCR and normalized to housekeeping gene *RPLP0*.

**E-F,** Box and whiskers plot of log2 normalized read counts of baseline expression of TNF and IFN Receptor genes (E) and *MAP3K7* (F) in sensitive (n=6) and insensitive GSCs (n=6) measured by RNAseq. Whiskers show minimum and maximum values within group. Boxes indicate median, upper and lower quartiles. **G,** Barplot of fold cell expansion of G166, G26 and G7 treated for 4 days with indicated drugs. All error bars show mean +/- SD of 3 biological replicates. p-values from t-test comparison with DMSO treatment are shown with ****p<0.0001, ***p<0.001, **p<0.01, *p<0.05, ns = not significant.

**­­­­­­­
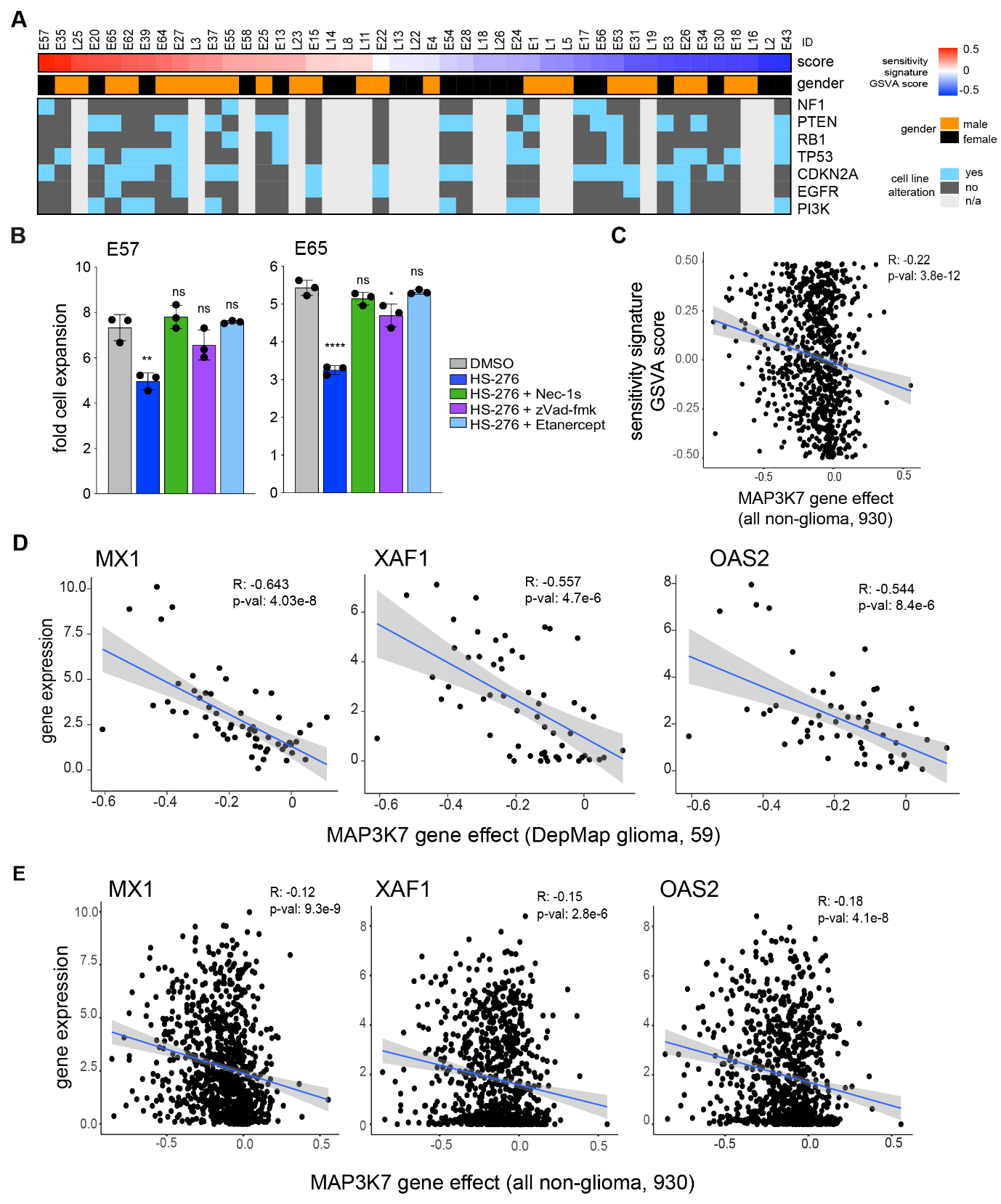
Figure S6. Sensitivity signature does not associate with known genetic alterations in GSCs and correlates with dependency on MAP3K7 gene function in non-glioma cell lines**. **A**, Heat map of GSVA sensitivity signature score, gender and genetic alterations in GCGR GSC lines. ID, GCGR patient ID. n/a, data not available. **B**, Barplot of fold cell expansion of GCGR-GSCs E57 and E65 treated for 4 days with indicated drugs. All error bars indicating mean +/- SD of 3 biological replicates. p-values from t-test comparison with DMSO treatment are shown with ****p<0.0001, **p<0.01, *p<0.05, ns = not significant. **C**, Scatter plot of MAP3K7 gene knockout effect against sensitivity signature GSVA score from gene expression data in 930 non-glioma cell lines from DepMap. **D-E**, Scatter plot of MAP3K7 gene knockout effect against RNAseq gene expression of interferon-stimulated genes MX1, XAF1 or OAS2 in 59 DepMap glioma (D) and 930 non-glioma cell lines (E). Person correlation R- and p-values are shown in each plot, blue = regression line of linear model with 95% confidence interval (gray).


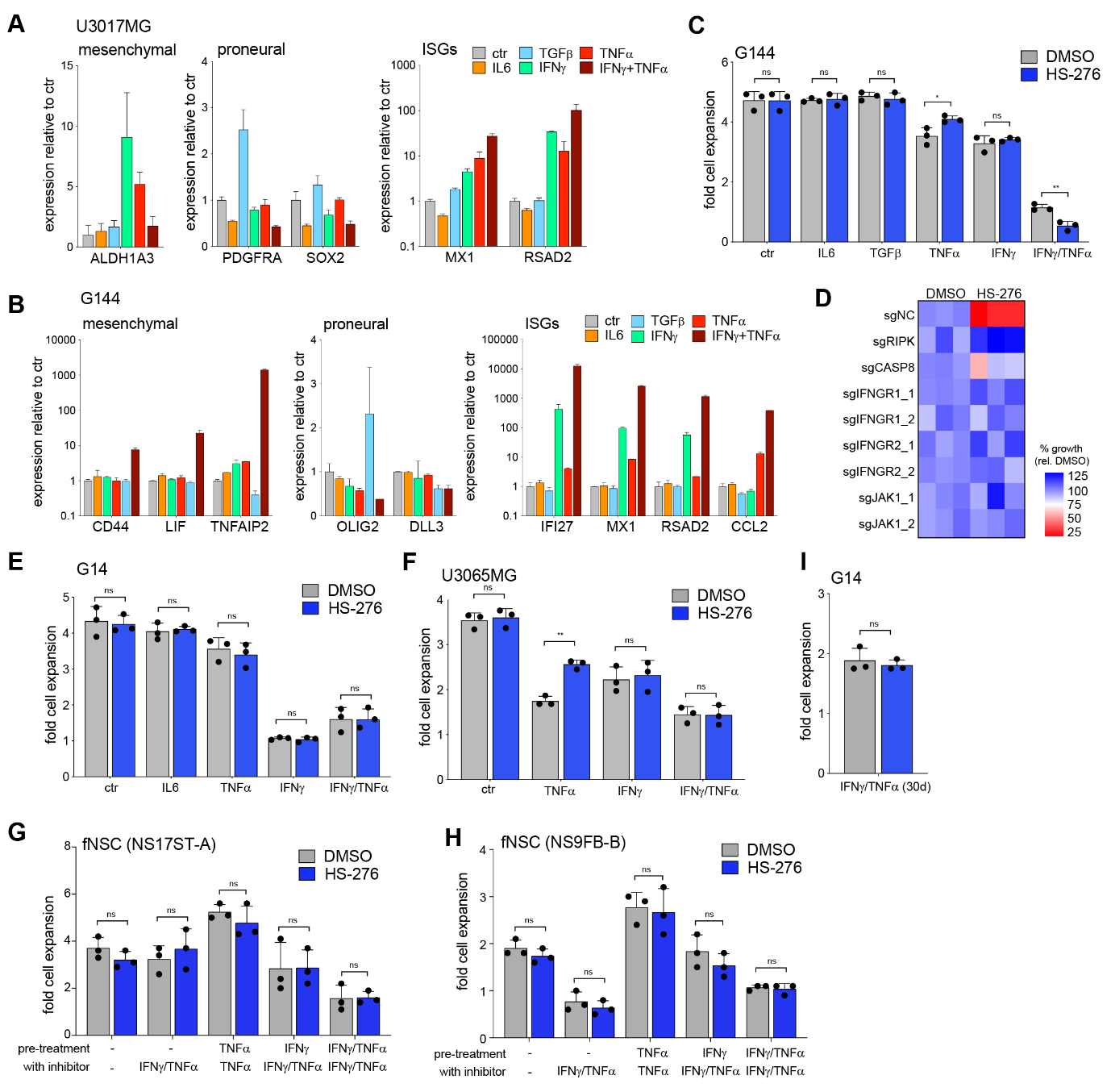


Figure S7: Combined IFNγ and TNFα pathway activation is required to sensitize GSCs to TAK1 inhibition. A-B, qPCR of ISGs, mesenchymal, or proneural marker gene expression after cytokine treatment in U3017MG (A) and G144 (B). Error bar indicates mean +/- SD of 2 technical replicates. D, Heatmap showing % growth of U3017MG cells with different IFN pathway mediator knockouts reprogrammed with IFNγ and TNFα followed by 4 days HS-276 treatment. Growth was normalized to mean value of respective DMSO control treatment and set to 100%. C,E-H, Fold cell expansion of G144 (C), G14 (E), U3065MG (F), fNSC NS17ST-A (G) and fNSC NS9FB-B (H) cells pre-treated for 3 days with indicated cytokines followed by 4 days of DMSO or HS-276. I, Fold cell expansion of G14 cells pre-treated for 30 days with IFNγ and TNFα followed by 4 days of DMSO or HS-276. All error bars indicating mean +/- SD of 3 biological replicates (unless stated otherwise). p-values from t-test comparison with DMSO treatment are shown with ****p<0.0001, ***p<0.001, **p<0.01, *p<0.05, ns = not significant.
