# Supplementary figures and images for: TAK1 inhibition leads to RIPK1-dependent apoptosis in immune-activated cancers
Source: Cell Death Dis. 2024 Apr 17;15(4):273. doi: 10.1038/s41419-024-06654-1 (PMC11024179; doi:10.1038/s41419-024-06654-1)

**Fig. 1D**

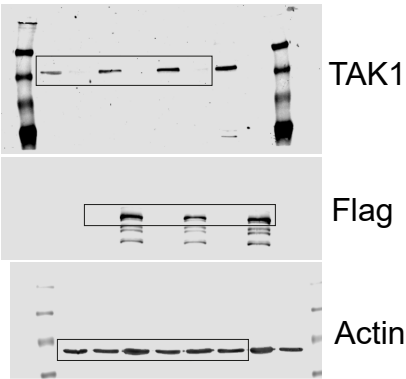

**Fig. 2A**

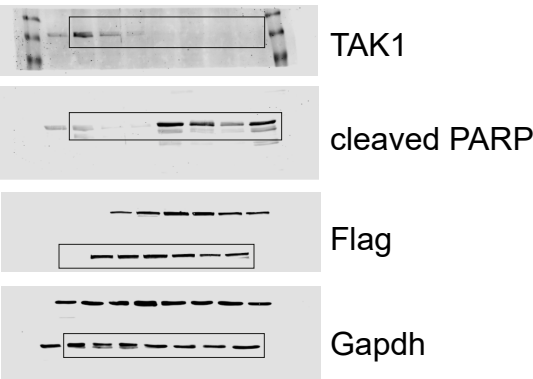

**Fig. 3B**

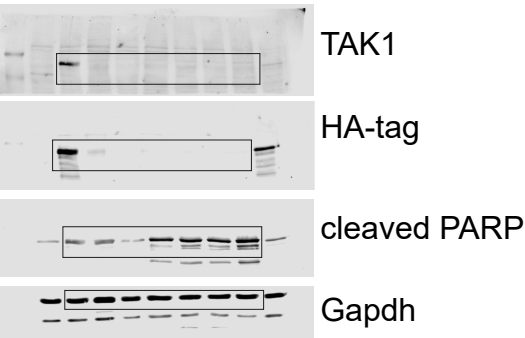

**Fig. 2F**

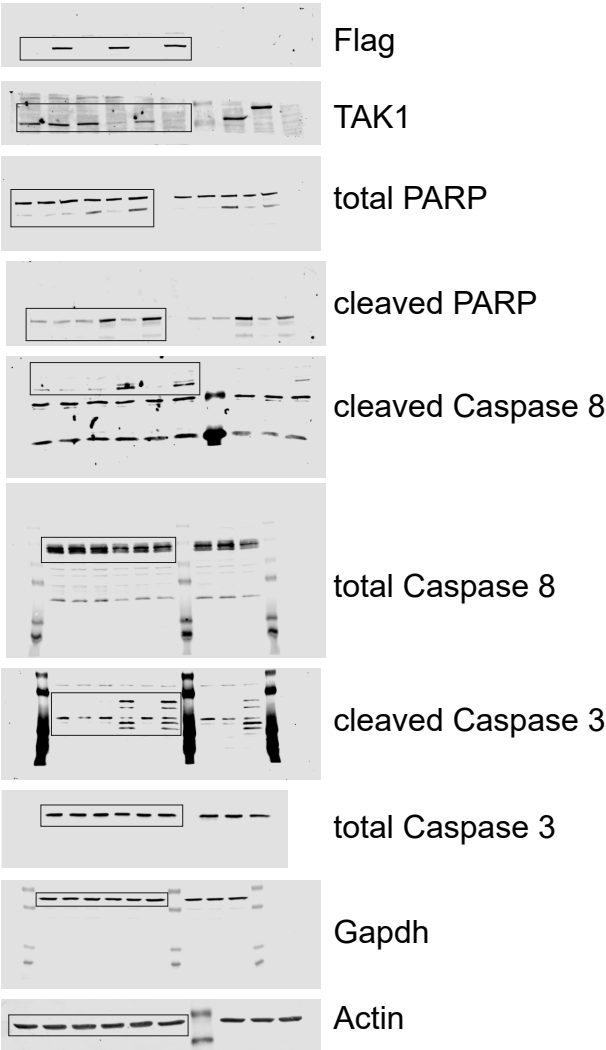

**Fig. 3D**

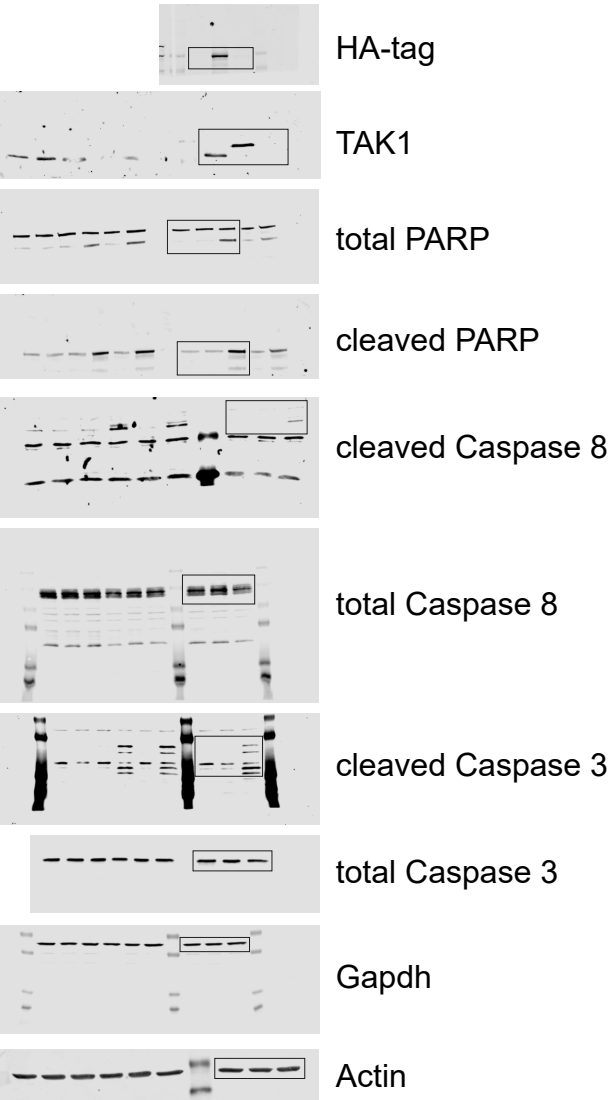

Fig. 4H

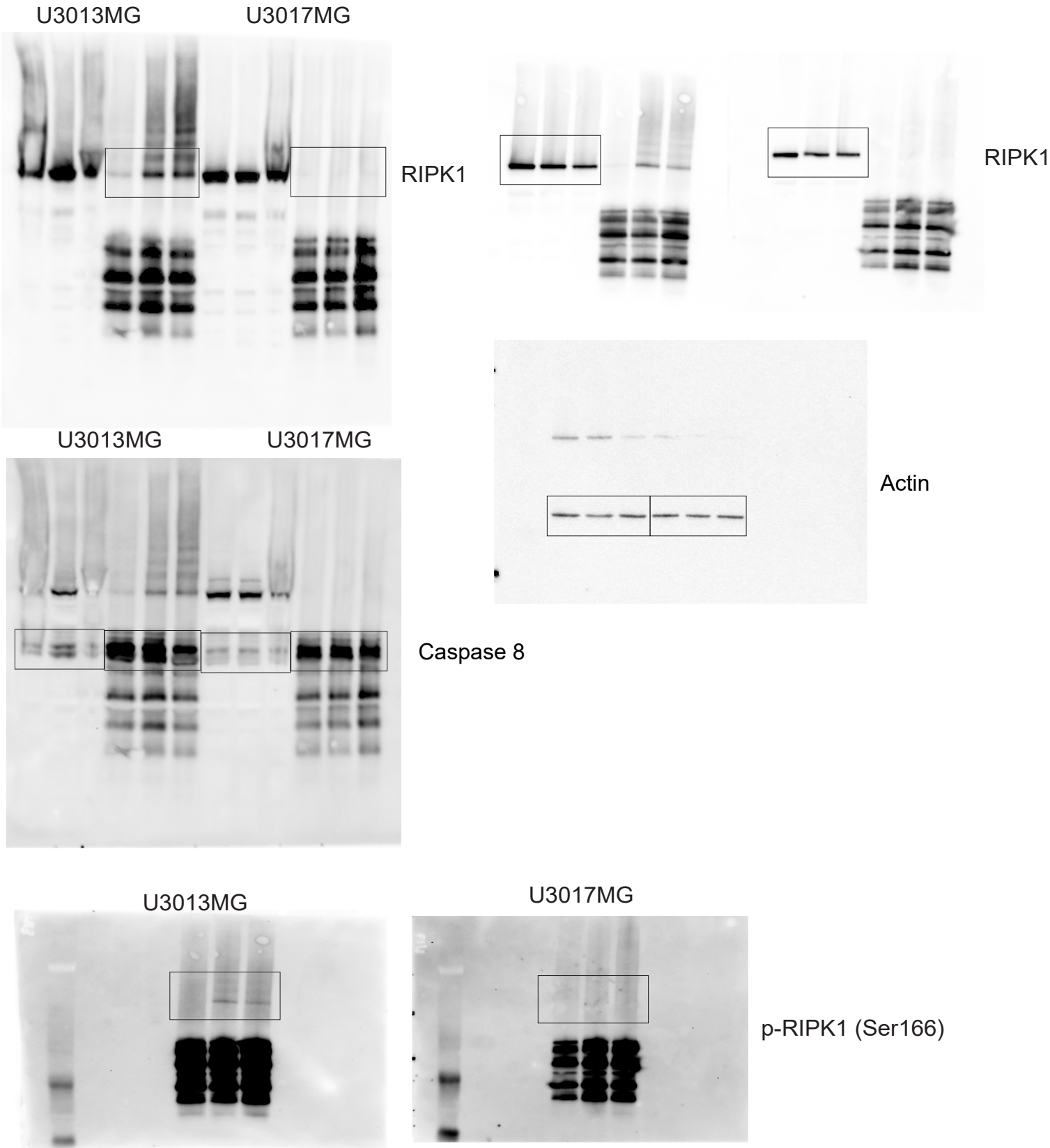

**Fig. S1C**

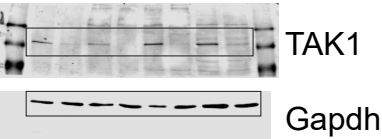

**Fig. S2D**

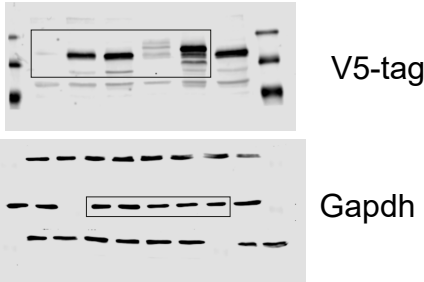

**Fig. S2A**

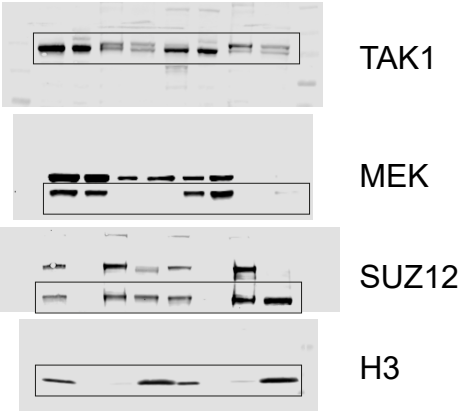

**Fig. S2A**

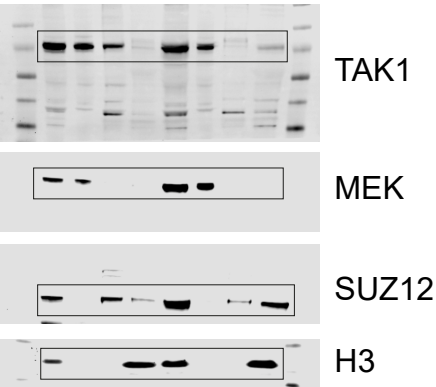

**Fig. S3A**

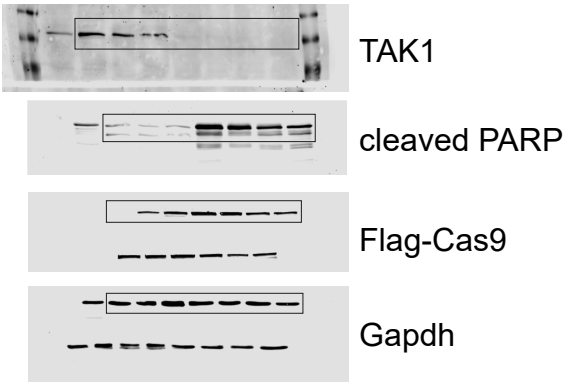

**Fig. S3D**

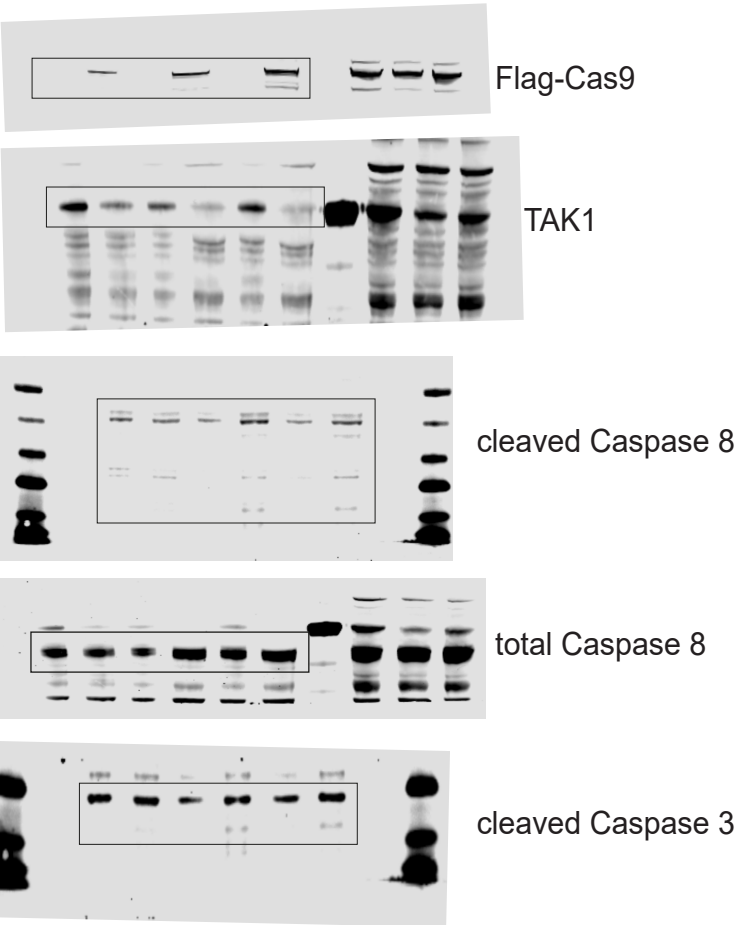

**Fig. S3D**

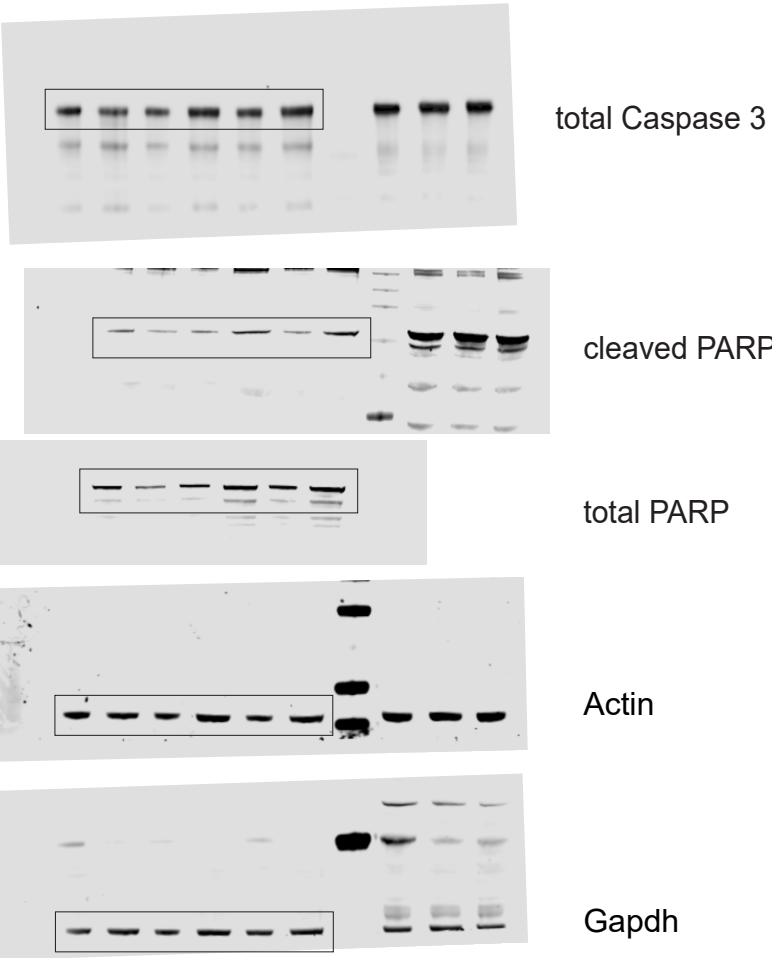

**Fig. S3C**

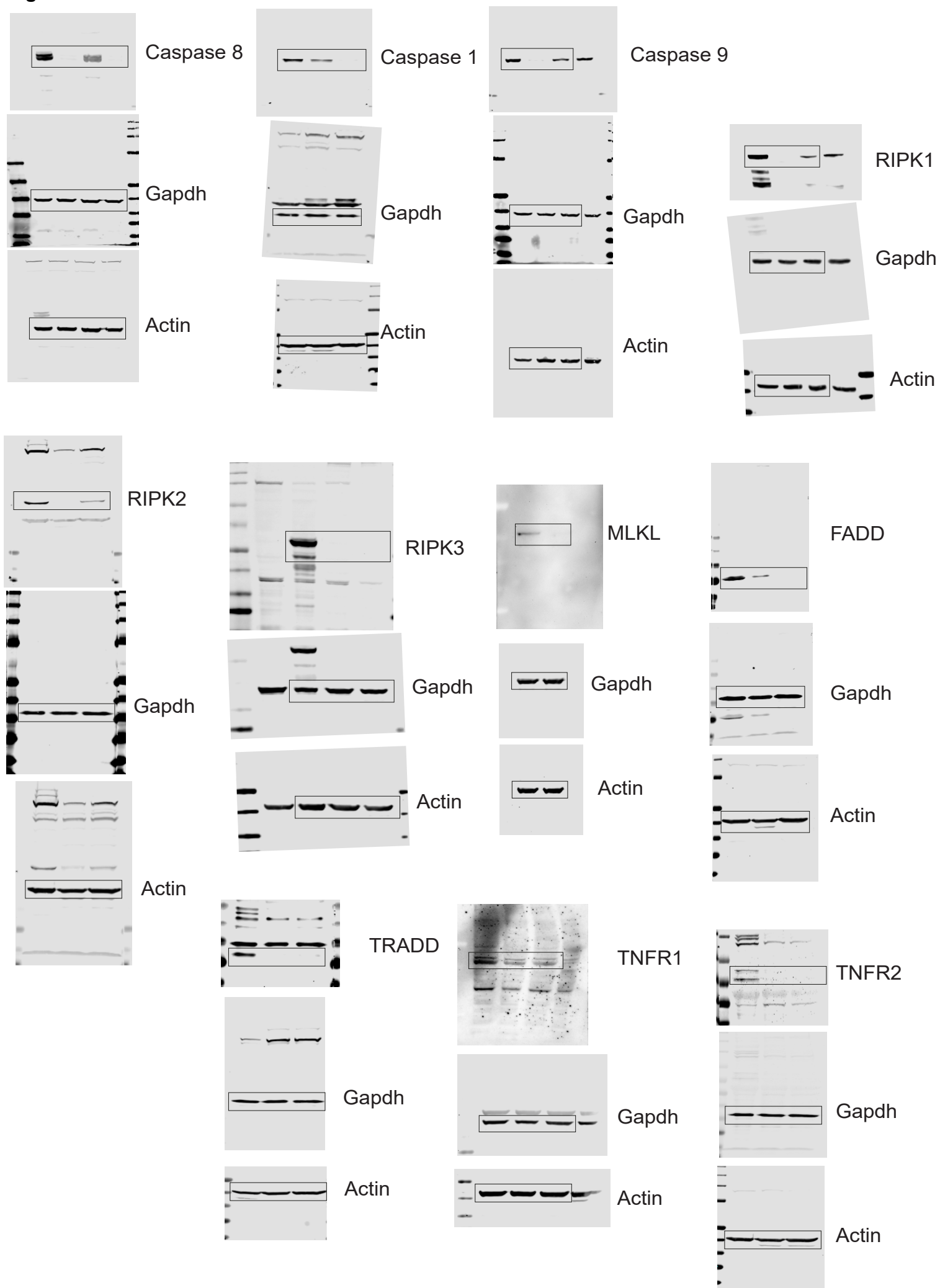

**Fig. S4D**

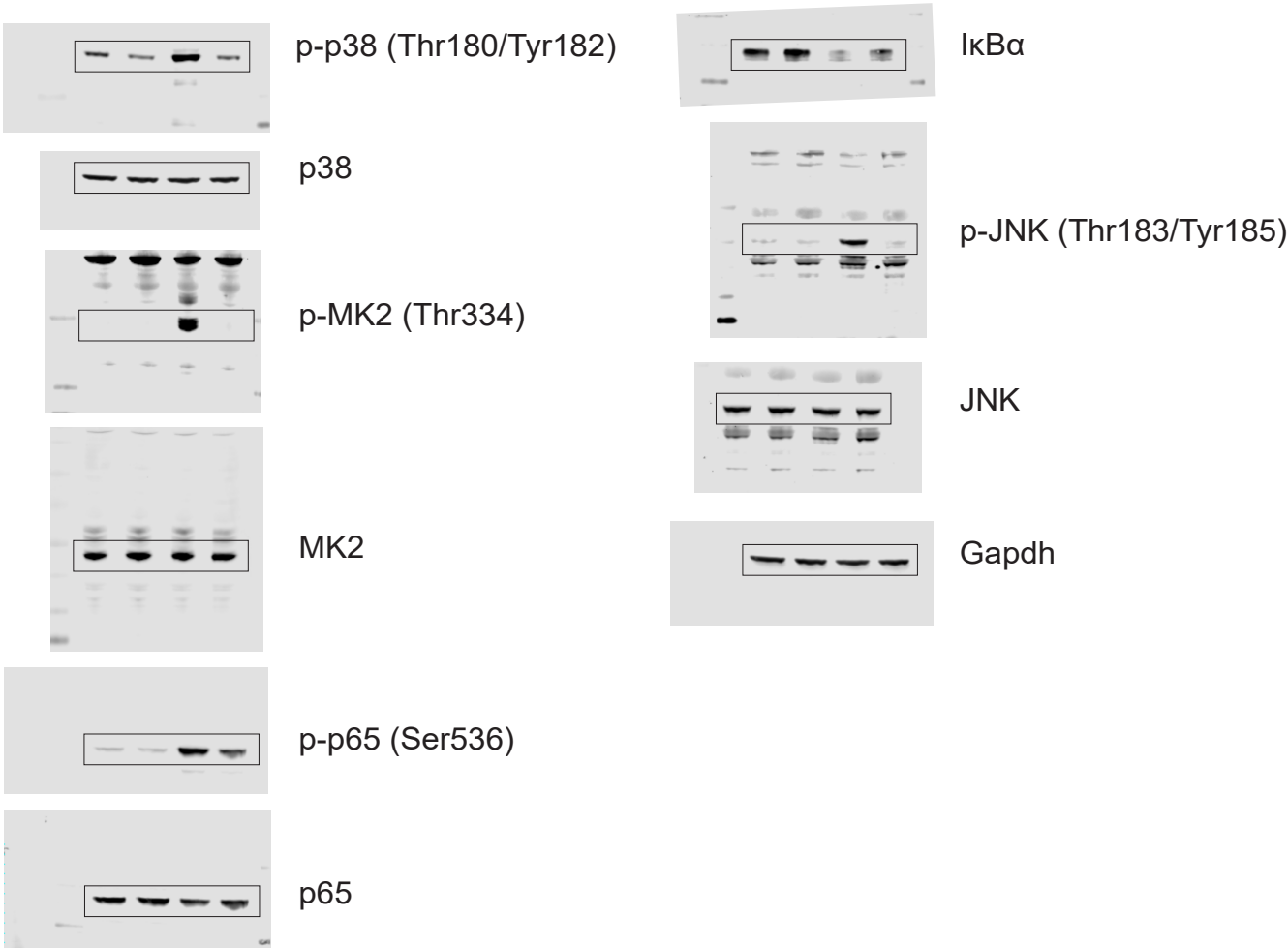

**Fig. 3I**

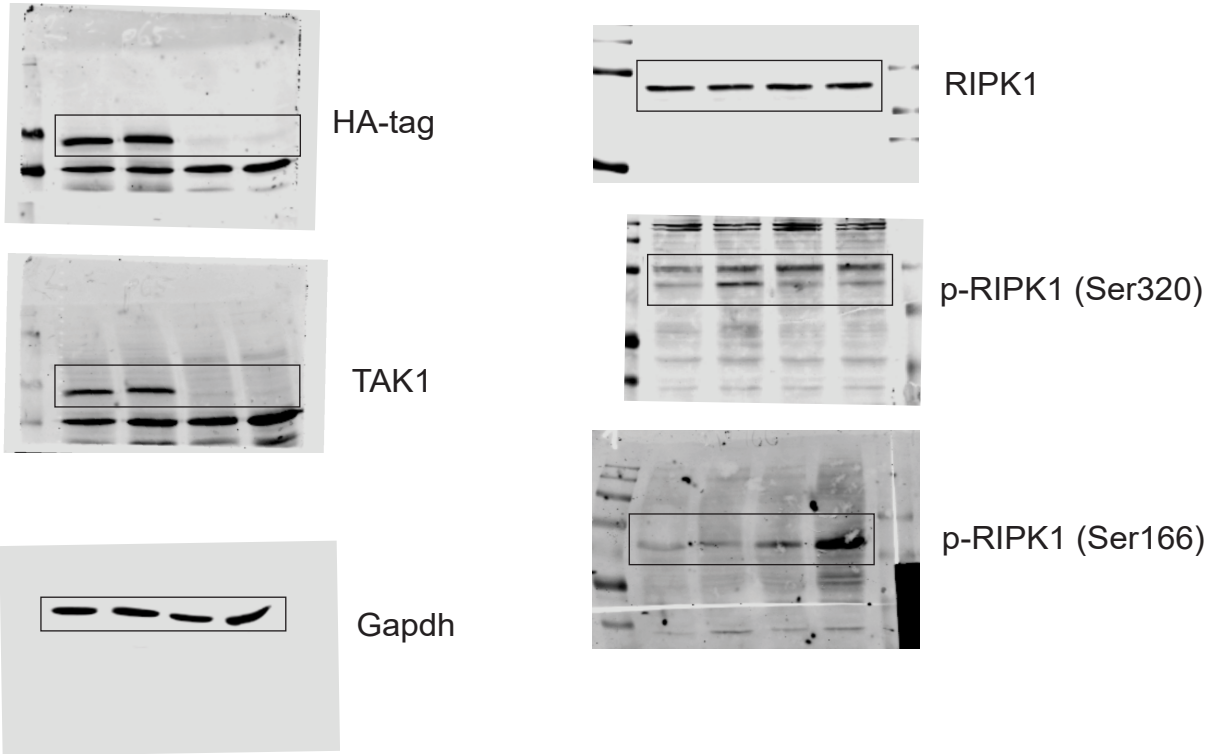

Supplement: Supplementary file 2 — Supplemental Information Western blots [file 41419_2024_6654_MOESM2_ESM.pdf]
